# Harnessing the power of marine terpenoids against diabetes-associated oxidative stress

**DOI:** 10.3389/fnut.2025.1651804

**Published:** 2025-08-06

**Authors:** Derren David Christian Homenta Rampengan, Juan Alessandro Jeremis Maruli Nura Lele, Raffaele Romano, Nurpudji Astuti Taslim, Raymond Rubianto Tjandrawinata, Antonello Santini, Fahrul Nurkolis

**Affiliations:** ^1^Faculty of Medicine, Universitas Sam Ratulangi, Manado, Indonesia; ^2^Clinical Clerkship Program, UKI Hospital East Jakarta, Faculty of Medicine, Universitas Kristen Indonesia (UKI), Jakarta, Indonesia; ^3^Department of Agricultural Sciences, University of Naples Federico II, Naples, Italy; ^4^Division of Clinical Nutrition, Department of Nutrition, Faculty of Medicine, Hasanuddin University, Makassar, Indonesia; ^5^Center for Pharmaceutical and Nutraceutical Research and Policy, Faculty of Biotechnology, Atma Jaya Catholic University of Indonesia, Jakarta, Indonesia; ^6^Department of Pharmacy, University of Napoli Federico II, Naples, Italy; ^7^Institute for Research and Community Service, State Islamic University of Sunan Kalijaga (UIN Sunan Kalijaga), Yogyakarta, Indonesia; ^8^Master of Basic Medical Science, Faculty of Medicine, Universitas Airlangga, Surabaya, Indonesia; ^9^Medical Research Center of Indonesia, Surabaya, Indonesia

**Keywords:** marine terpenoids, oxidative stress, type 2 diabetes mellitus (T2DM), antioxidant, pancreatic β-cell, hyperglycemia, diabetic complications, redox signaling

## Abstract

Diabetes mellitus (DM), particularly type 2 diabetes (T2DM), remains a significant global health concern, driven largely by oxidative stress-induced damage. Marine terpenoids, bioactive compounds extracted from diverse marine organisms such as algae, sponges, and corals, present promising antioxidant and antidiabetic potential. This review systematically evaluates the chemical diversity, biological sources, and mechanisms of action of marine terpenoids in mitigating diabetes-associated oxidative stress. Marine terpenoids exhibit potent antioxidant capabilities via radical scavenging, modulation of cellular antioxidant defenses, regulation of redox-sensitive pathways such as Nrf2/ARE and NF-κB, metal chelation, and pro-oxidant enzyme inhibition. Preclinical studies underscore their efficacy in reducing hyperglycemia, enhancing insulin sensitivity, preserving pancreatic β-cell function, and protecting against diabetic complications, including nephropathy and cardiovascular diseases. Despite the promising preliminary results, further studies addressing bioavailability, pharmacokinetics, long-term safety, and sustainability are imperative to establish marine terpenoids as viable therapeutic options for diabetes management.

## 1 Introduction

The global prevalence of diabetes mellitus (DM), in particular type 2 diabetes mellitus (T2DM), is increasing continuously and has been predicted to have more than 592 million cases by 2035 (1). The complicated consequences of DM and its increasing prevalence continue to be major challenges in healthcare management (2). Oxidative stress as one of the many complicated mechanisms implicated in the etiology of DM and its complications is the center of focus in this review on the use of marine terpenoids as novel antioxidant and antidiabetic agents. Considering the vast and yet unexplored marine biodiversity, marine pharmacology is emerging as a new era in antidiabetic drug discovery.

DM is a condition of chronic hyperglycemia from altered insulin secretion, action or both, that disturbs metabolism of carbohydrate, fats, and protein. Pathologically in diabetes, oxidative stress occurs because of a greater generation of ROS than ROS-scavenging enzymes can remove. An imbalance in the redox status of cells produces lipid peroxidation, protein oxidation, DNA injury and activates various signal pathways such as the NF-κB and Nrf2/ARE signaling, all resulting in insulin resistance, pancreatic β-cell dysfunction, and various micro- and macrovascular complications of DM such as neuropathy, nephropathy, and hepatic damage (3).

Marine environments represent the majority of the Earth’s surface. Marine biotas generate structurally diversified, biologically active secondary metabolites. Among the diverse compounds extracted from marine organisms, terpenoids from marine algae, sponges, corals and microorganisms are the most extensive family of natural products with various carbon skeleton types (e.g., monoterpenes, sesquiterpenes, diterpenes, sesterterpenes, and triterpenes). Terpenoids have multiple biological effects, including antioxidant, anti-inflammatory, and antidiabetic effects (4, 5). Compared to terrestrial organisms, marine organisms can produce novel terpenoid structures, which enhance their pharmacological activity. Yet, the evidence for their antidiabetic mechanism of action remains limited. Hence, this review addresses the research question: What is the evidence for the antioxidative and antidiabetic activities of marine terpenoids and their molecular mechanisms of action?

Here, we summarize the chemical and structural diversity, source organisms, and mechanism of action of marine terpenoids on diabetes, along with their antioxidant effect through direct free-radical scavenging, redox-sensitive signal-network modulation, and induction of intracellular ROS scavenger enzyme expression. The review covers *in vitro* and *in vivo* evidence regarding the ability of marine terpenoids to decrease hyperglycemia, to maintain the function of pancreatic β-cells, and to scavenge for ROS and oxidative damage. Several representative marine terpenoids that possess dual antioxidant and antidiabetic activities, such as sargaquinoic acid, fucosterol, and halimane diterpenoids, are discussed. In addition, the ability of marine terpenoids to prevent or cure diabetes complications, for example, neuropathy, nephropathy, vascular and hepatic complications, are highlighted. Also, we briefly cover the pharmacokinetic, safety and formulation of marine terpenoids in clinical trial and application, along with current limitations and future studies required in this area.

Consistent with most pharmacological research on marine-sourced bioactive compounds, research on marine terpenoids in diabetic management is still at a relatively initial stage, with limited data gathered from *in vitro* cell culture studies. Current evidence obtained with two polysaccharides fucoidan and bromophenols from marine brown algae indicates a potential role of these compounds in the treatment of diabetic complications (6, 7), although a thorough elucidation of the pharmacological mechanism, sustainable production and availability are required for development as therapeutic agents. These barriers necessitate an interdisciplinary approach involving pharmacology, biochemistry, and marine biotechnology to advance this field.

Overall, this review provides a comprehensive overview of the potential of marine terpenoids in combating oxidative stress associated with DM, particularly T2DM. The primary focus of this review includes an evaluation of the chemical diversity and biological sources of marine terpenoids, elucidation of their antioxidants and antidiabetic mechanisms of action involving direct radical scavenging, modulation of redox-sensitive signaling pathways such as Nrf2/ARE and NF-κB, and induction of cellular antioxidant enzyme expression. Additionally, we summarize preclinical evidence supporting the ability of marine terpenoids to reduce hyperglycemia, maintain pancreatic β-cell function, and prevent or mitigate diabetic complications such as neuropathy, nephropathy, and vascular and hepatic disorders. Finally, this review addresses translational challenges, highlighting issues related to bioavailability, extraction methods, formulation strategies, and further research required to ensure the safety and effectiveness of marine terpenoids as promising therapeutic agents for the management of diabetes and its associated complications.

## 2 Oxidative stress in diabetes mellitus

**Figure 1:**
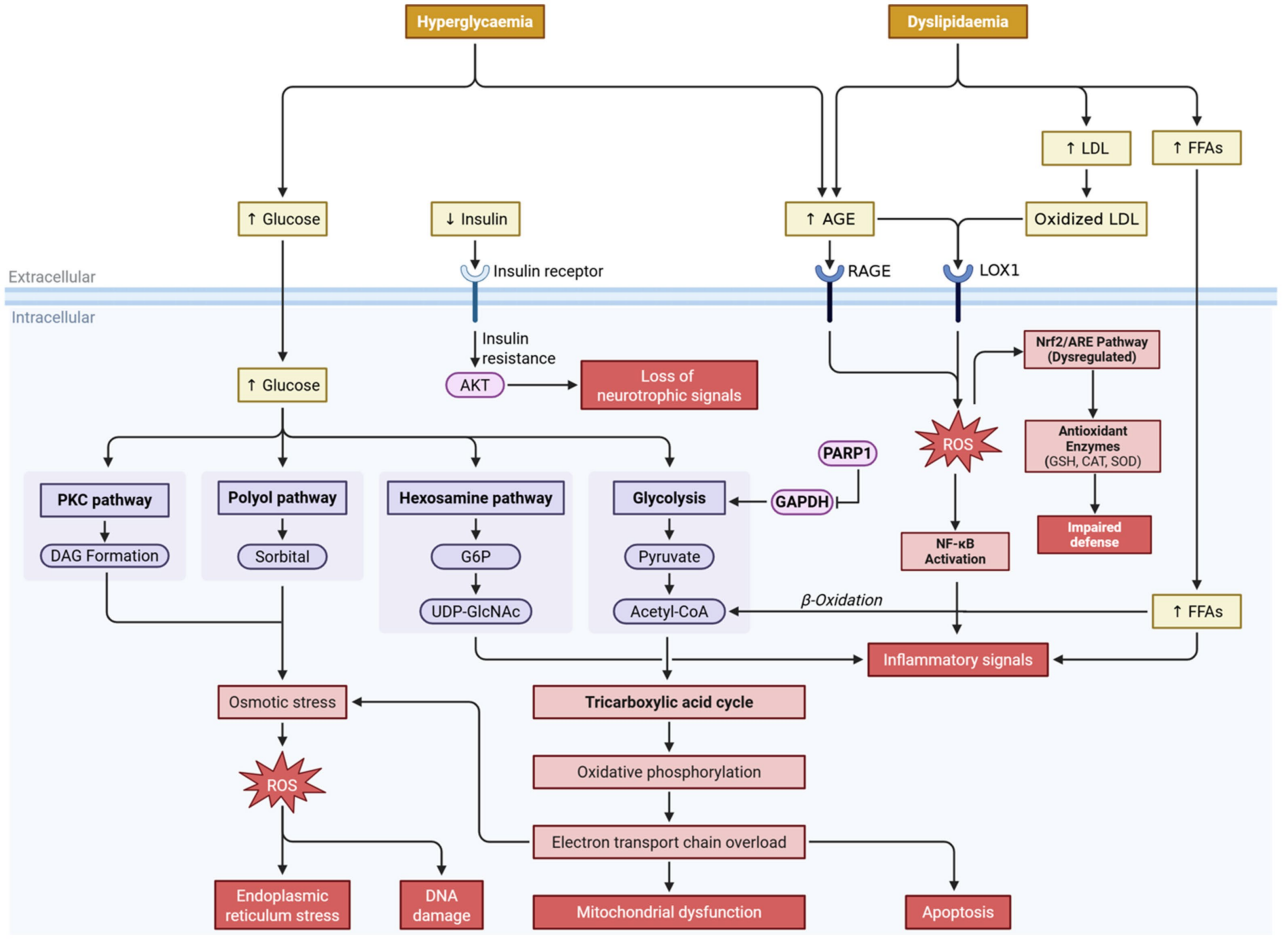
Mechanistic overview of hyperglycemia- and dyslipidemia-induced oxidative stress in diabetes mellitus. Chronic hyperglycemia and dyslipidemia synergistically trigger multiple metabolic pathways—including PKC, polyol, hexosamine, and AGE pathways—that converge in the overproduction of reactive oxygen species (ROS). Elevated ROS levels promote mitochondrial dysfunction, endoplasmic reticulum (ER) stress, DNA damage, and apoptosis, aggravating diabetic complications. Meanwhile, oxidized LDL and AGEs activate RAGE and LOX1 receptors, further enhancing oxidative stress and NF-κB-mediated inflammation. Dysregulation of the Nrf2/ARE antioxidant pathway impairs cellular defense mechanisms. The resulting oxidative-inflammatory cascade contributes to β-cell dysfunction, insulin resistance, and progressive micro- and macrovascular complications.

Oxidative stress results in metabolic dysfunction and tissue damage that contribute to the onset and progression of DM. Evidence indicates that increased ROS production due to an imbalance of ROS production and antioxidant defenses lead to disrupted insulin signaling, mitochondrial dysfunction, and progressive β-cell deterioration, which are hallmarks of the chronic complications of the disease (Figure 1) (8). Accumulation of oxidative damage products as a result of depletion of endogenous antioxidants such as glutathione (GSH), catalase (CAT) and superoxide dismutase (SOD) lead to metabolic dysfunction and tissue damage (9–11).

Individuals with diabetes develop increased oxidative stress which accelerates the formation of advanced glycation end-products (AGEs) and lipid peroxidation, resulting in cellular senescence and eventual organ dysfunction (12). This has an impact on organs such as vascular, neural and renal tissues that are more sensitive to diabetic complications. The increased oxidative stress will initiate nuclear factor-kappa B (NF-κB) and Nrf2/ARE pathways, which results in inflammation and a greater oxidative insult, leading to insulin resistance and β-cell deterioration. Therefore, therapeutic applications that involve the use of bioactive compounds with both antioxidant and ROS scavenger properties are considered to be a viable option in the treatment of diabetes (9, 11).

Hyperglycemia in diabetes stimulates several pro-oxidative pathways that are involved in ROS production and tissue damage, including glycolytic, hexosamine, PKC, polyol, and AGE pathways. Hyperglycemia in the glycolytic pathway can result in increased flux in glycolytic intermediates to alternative pathways. An example of this is the polyol pathway, in which glucose is reduced to sorbitol by aldose reductase, resulting in NADPH depletion, thus reducing antioxidant defenses. Elevated diacylglycerol levels stimulate the PKC pathway leading to reduced vasorelaxation, inflammation, and superoxide radical formation. Increased AGE levels enhance ROS production and pro-oxidant signaling (10).

The hexosamine pathway is also activated by hyperglycemia, leading to protein modifications and increasing cellular stress by activating pro-oxidant signaling pathways. Activation of poly-ADP-ribose polymerase 1 (PARP1) in response to hyperglycemia will cause the inhibition of glyceraldehyde-3-phosphate dehydrogenase (GAPDH) activity due to depletion of glycolytic intermediates and resulting in an accumulation of upstream metabolites. The accumulation of glycolytic intermediates increases the flux of these intermediates into the AGE and PKC pathways, contributing to metabolic defects and increased ROS production. Accumulation of glyceraldehyde-3-phosphate (G3P) will be converted to methylglyoxal, which is the most potent precursor for AGEs, and directly contribute to increased oxidative stress and tissue injury. Moreover, G3P accumulation will also lead to increased diacylglycerol formation, activating PKC and altering cellular homeostasis, leading to vascular complications. Additionally, the accumulation of glycolytic intermediates will also flux to the polyol and hexosamine pathways and contribute to greater ROS production and metabolic toxicity, thus initiating the progression of vascular complications (10).

This metabolic-oxidative cascade in diabetes promotes microvascular and macrovascular complications in diabetic patients and is the prime target for therapeutic intervention of this disease. High oxidative stress levels can damage biological molecules such as lipids, proteins, and DNA. Increased ROS can result in increased lipid peroxidation in cellular membranes and the subsequent release of aldehyde products. Aldehyde products from lipid peroxidation are toxic and can affect cell integrity and result in cellular apoptosis, in particular pancreatic β-cells. Protein oxidation leads to inactivation of key proteins that are involved in glucose homeostasis. Protein damage impairs tissue regeneration and the reparation of cell repair and regeneration. DNA damage due to oxidative stress can cause genetic mutations that result in cancer and decreased cell division and may contribute to vascular and renal complications in diabetes. Furthermore, increased ROS also activates NF-κB signaling, which increases pro-inflammatory cytokine expression. The activated NF-κB pathway enhances expression of adhesion molecules and contributes to further metabolic complications. Nrf2/ARE plays a protective role by inducing antioxidant enzymes and phase II detoxification enzymes. However, this antioxidant pathway is dysregulated during diabetes and creates a need for potential therapeutic intervention to promote its activation (9, 11).

Marine terpenoids and carotenoids, such as astaxanthin and fucoxanthin, display *in vivo* antioxidant activity. They can increase the activities of endogenous antioxidant enzymes and alleviate ROS-mediated cellular damage. Additionally, these compounds possess hypoglycemic effects and contribute to the amelioration of diabetes. Astaxanthin inhibits oxidative stress-related apoptosis in β-cells, enhances CAT and SOD activities, and increases GSH levels. Fucoxanthin demonstrates antioxidant potential and beneficial glycemic control and also exerts many cardiovascular benefits. The potential use of astaxanthin in the treatment of diabetes is particularly relevant because of its superior antioxidant capabilities compared to other carotenoids. Marine terpenoids such as fucosterol and sargaquinoic acid from brown algae, *Sargassum fusiforme*, have exhibited the improvement of insulin signaling, as well as hypoglycemic effects (9, 13).

These marine compounds can regulate redox-sensitive signaling pathways such as Nrf2/ARE activation and NF-κB inhibition, which make these bioactive agents’ effective modulators in controlling not only primary metabolic defects in diabetes, but also in the prevention and treatment of secondary complications. While preclinical trials suggest these compounds as potential therapeutic treatments, translation is difficult due to limited bioavailability, poor metabolism, and/or limited applicability. Studies on dehydroeburicoic acids are effective in managing hyperglycemia, even more than standard antidiabetic medications, but the translation to human studies remains unexplored. This suggests that while marine terpenoids hold significant potential in controlling and treating diabetes, human studies are imperative. It is important to recognize that most findings have been obtained from short-term experimental animal models. Longitudinal human studies are required to determine the long-term safety profile, applicability to diverse populations, and effectiveness in the treatment of chronic conditions such as diabetes (11, 13).

Furthermore, to optimize the utilization of marine terpenoids, further exploration into the methods of extraction, isolation, purification, dosing strategies, and potential combinatorial interventions may be required. More investigations will be needed to identify subpopulations who will respond to intervention therapies based on marine terpenoids, as well as the combination of marine terpenoids with available standard therapies to better optimize patient outcomes. The effectiveness of these bioactive agents will depend on the standardization of dosing and formulations. The ability of marine terpenoids to effectively control glycemia may also depend on the individual’s overall health and dietary habits. To ensure the use of this bioactive compound is safe and reliable, it will be necessary to identify those populations most at risk of toxicity or allergenicity in order to make decisions about whom marine terpenoids may not be suitable as an antidiabetic agent.

## 3 Marine terpenoids: classification, sources, and chemistry

**Figure 2:**
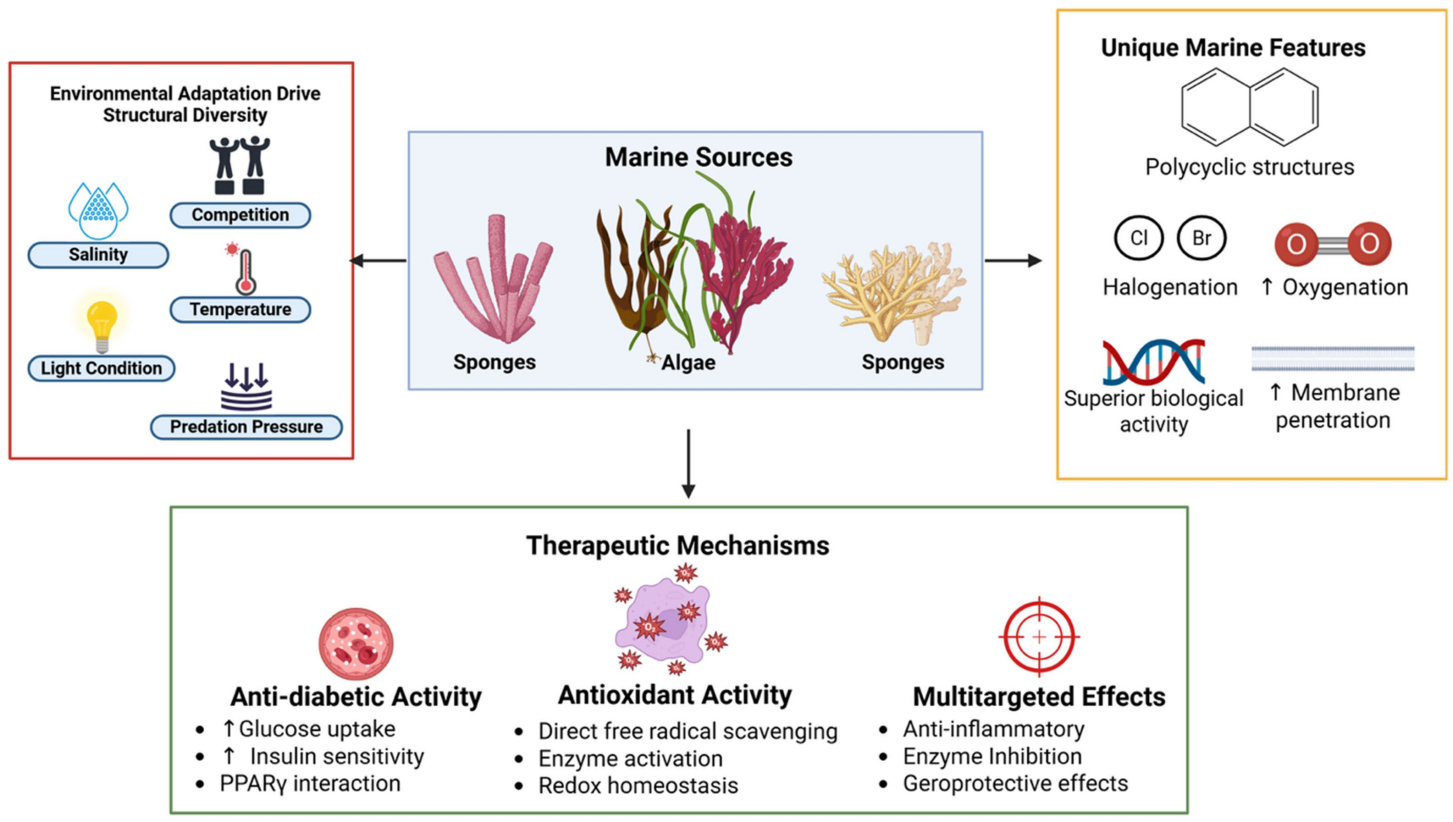
Marine terpenoids as promising therapeutic agents, for instance, structural origins, unique features, and biological mechanisms. Environmental stressors in marine ecosystems, including salinity, light, temperature, and predation pressure, drive the structural diversification of bioactive terpenoids derived from marine algae and invertebrates such as sponges and corals. These compounds exhibit distinctive chemical features, including polycyclic backbones, halogenation, and high oxygenation levels, which confer enhanced biological activity and membrane permeability. Therapeutically, marine terpenoids demonstrate potent anti-diabetic effects by improving glucose uptake and insulin sensitivity through PPARγ interaction, robust antioxidant action via radical scavenging and enzyme activation, and broad multitargeted effects such as anti-inflammatory, enzyme inhibitory, and geroprotective properties—highlighting their potential in addressing complex metabolic disorders like diabetes mellitus.

Marine terpenoids are diverse classes of natural products with potential therapeutic applications for oxidative stress and diabetes (Figure 2). This classification of marine terpenoids will reveal their diversity, biological source, and chemical features that are useful for drug discovery in the realm of natural products.

### 3.1 Classification of terpenoids (monoterpenes, sesquiterpenes, diterpenes, etc.)

Marine algae, particularly brown seaweeds (Phaeophyceae), are one of the richest sources of terpenoids. Brown algae (e.g., Sargassum, Ecklonia, Undaria) produce an array of diterpenes, triterpenes, sterols (like fucosterol), and carotenoids (fucoxanthin) with significant bioactivities (10, 14). The structural variability of algal terpenoids arises from both genetic factors and environmental conditions such as light, temperature, nutrients, and salinity (15). For instance, many brown algae synthesize halogenated terpenes (e.g., brominated sesquiterpenes) as a survival strategy, in which the halogenation and polycyclic structures may enhance their ability to neutralize free radicals and adapt to harsh marine environments (6). A notable example is fucosterol, a C_29_sterol from Sargassum and Undaria, which has shown potent antidiabetic effects by improving insulin sensitivity and modulating redox inflammatory pathways (16, 17). Another is sargaquinoic acid, a meroterpenoid (terpene-benzoquinone) from Sargassum species, with a conjugated quinone structure that confers strong electron-donating (antioxidant) capacity and α-glucosidase inhibitory potency (18). These compounds highlight how algae leverage unique structural features (e.g., quinone moieties, extensive conjugation, halogen substituents) to achieve dual antioxidant and hypoglycemic functions. Algal terpenoids can also act synergistically with other algal metabolites like polysaccharides and polyphenols. For example, whole extracts from brown algae sometimes inhibit carbohydrate-hydrolyzing enzymes more effectively than isolated terpenoids alone (19). However, the study of such synergistic interactions is still limited.

A practical challenge with algae as a source is the variability in metabolite yield and composition. Seasonal and environmental fluctuations can change the terpenoid content in wild algae, complicating mass production and standardization (20). Some advances in marine biotechnology address this: controlled algal aquaculture and metabolic engineering are being explored to produce key terpenoids consistently (21, 22). For instance, metabolic engineering can upregulate the biosynthetic pathways for target terpenoids, while photobioreactor cultivation of algae provides a steady biomass supply (21). Though promising, these approaches require significant investment and optimization.

Importantly, marine algae appear to have evolved more multi-targeted antioxidant mechanisms compared to terrestrial plants, possibly because of intense competition and stress in the ocean. It’s hypothesized that algal terpenoids are “designed” by natural selection to protect against a broad spectrum of stressors, thus showing higher efficacy in mitigating oxidative stress and metabolic dysfunction in experimental models (23). While this hypothesis is compelling, more research is needed to correlate specific structural traits of algal terpenoids with their bioactivity profiles (i.e., structure-activity relationships across a broader range of algal species) (24).

Sponges (Porifera) and soft corals are prolific producers of complex terpenoids, including sesterterpenes (C_25_) and diterpenoids that are rarely found on land. Many sponge/coral terpenoids belong to unique skeletal classes such as halimane diterpenoids, cigualane and spirolane triterpenes, and various meroterpenoids, often featuring halogen atoms and intricate ring systems (25). These structural novelties yield potent radical scavenging and enzyme-modulating activities (26, 27). For example, certain halimane diterpenoids isolated from sponges exhibit pronounced antioxidant effects by virtue of their bicyclic skeleton with multiple hydroxyls, which facilitate both free radical quenching and inhibition of pro-oxidant enzymes (like NADPH oxidases) (28). Sponges can not only neutralize ROS but also help restore antioxidant enzyme levels in oxidative stress conditions, making their metabolites intriguing candidates for treating oxidative stress-related diseases (28). Similarly, soft corals have yielded terpenoids that display anti-inflammatory and antioxidant effects dependent on their structures (27). A notable class from corals are briaranes (diterpenoids) and from sponges sipholane triterpenes, which some of these have been identified as protein tyrosine phosphatase 1B (PTP1B) inhibitors and α-glucosidase inhibitors, targeting key enzymes involved in diabetes and insulin resistance (29, 30). For instance, sargachromenol and sargaquinoic acid (actually from a brown alga Sargassum, but similar quinone-terpenoids) showed IC_50 ~42 μM and ~96 μM against α-glucosidase, respectively, and ~12 μM against PTP1B, indicating significant enzyme inhibition activity relevant to postprandial hyperglycemia control.

One limitation of sourcing terpenoids from sponges/corals is supply and sustainability. Harvesting these slow-growing marine invertebrates at scale is ecologically and logistically challenging, and their metabolite profiles can vary by species and habitat (22, 31). Efforts such as sponge aquaculture and fermentation of symbiotic microbes are under investigation to provide sustainable production of sponge-derived terpenoids (22). Moreover, the complex chemical synthesis of these multi-ring terpenoids is often difficult due to stereochemical complexity (22). These issues necessitate innovative solutions (e.g., marine microbial culture or heterologous gene expression systems) to obtain sufficient quantities for drug development.

Beyond macroalgae, marine microalgae and phytoplankton are sources of important terpenoids like carotenoids. For example, the microalga *Haematococcus pluvialis* produces astaxanthin, a xanthophyll carotenoid (tetraterpenoid) responsible for the pink-orange pigmentation of salmon and shrimp (32). Astaxanthin’s structure (a C_40_carotene dione with hydroxyl and keto moieties on each end) enables it to insert into cell membranes and span both polar and nonpolar regions. This confers astaxanthin exceptional antioxidant capacity, able to quench free radicals within membranes and at membrane surfaces (32). Astaxanthin has demonstrated multiple antidiabetic actions. For instance, it scavenges ROS, protects β-cells from oxidative apoptosis, reduces lipid peroxidation, and even exerts anti inflammatory effects by modulating signaling pathways (33). In diabetic animal models, astaxanthin improved insulin sensitivity and pancreatic function, and in human studies it has shown benefits on glycemic control and blood pressure (34). Another microalgal terpenoid is β-carotene (from Dunaliella), though its antidiabetic impact is less pronounced than astaxanthin’s. Marine-derived sesquiterpene quinones from marine fungi and cyanobacteria also exist, but these are less explored in the context of diabetes.

In conclusion, marine-derived terpenoids, especially from brown algae, sponges, soft corals, and microalgae, exhibit significant antidiabetic potential through diverse mechanisms such as antioxidant activity, α-glucosidase and PTP1B inhibition, and insulin sensitization. Compounds like fucosterol, sargaquinoic acid, and astaxanthin demonstrate the ability to modulate redox balance and glucose metabolism, supported by unique structural features such as halogenation, quinone moieties, and extended conjugation. While environmental variability and sustainability issues pose challenges for large-scale production, particularly for sponge- and coral-derived terpenoids, advances in marine biotechnology, including controlled cultivation and metabolic engineering, offer promising solutions. Overall, marine terpenoids represent a structurally rich and mechanistically diverse class of compounds with strong potential for development into multifunctional therapeutics for diabetes mellitus.

### 3.2 Marine organisms producing terpenoids (algae, sponges, corals, etc.)

To better understand the pharmacological relevance of marine terpenoids in diabetes, it is important to examine the biological sources and structural uniqueness of these compounds. Tables 1, 2 provide a comparative overview of marine organisms that produce terpenoids and how these marine-derived compounds differ from their terrestrial counterparts. Table 1 provides a comparative overview of some key marine organisms, the representative terpenoids they produce, and features that distinguish them.

**Table 1:** Marine organisms as sources of terpenoids (examples and key features).

Marine source	Example terpenoids (class)	Unique structural features & adaptations
Brown algae (e.g., Sargassum, Ecklonia)	Fucosterol (triterpenoid sterol); sargaquinoic acid (meroterpenoid); fucoxanthin (carotenoid)	Halogenated and polycyclic diterpenes common; high oxygenation (quinones, epoxides). Terpenoids are often conjugated with phenolic motifs (meroterpenes) (6). Structural diversity driven by variable salinity, light, and predation pressure. Potent multi-target antioxidants and enzyme inhibitors in extracts (19)
Marine sponges (e.g., Callyspongia, Aplysina) & corals (soft corals)	Halimane diterpenoids; sesterterpenes (e.g., scalaranes); sipholenol triterpenes	Complex polycyclic frameworks often *absent in terrestrial analogs* (25); frequent halogenation (Br, Cl) of rings (25). These features enhance membrane permeability and are binding to protein targets. Noted for strong radical scavenging and PTP1B/α glucosidase inhibition activities (26, 46)
Microalgae & plankton (e.g., Haematococcus microalga)	Astaxanthin, β-carotene (tetraterpenoids)	Extended conjugated polyene chains with terminal polar groups (xanthophylls). Enable integration into lipid membranes and effective quenching of ROS *in situ*. Astaxanthin’s unique keto-carotenoid structure yields antioxidant activity superior to many terrestrial carotenoids (33) and broad benefits (e.g., improves antioxidant enzyme levels, insulin sensitivity in models)
Marine bacteria & fungi (symbionts or free-living)	(Varied terpenoids, e.g., isoprenoids in symbiotic bacteria)	Some marine microbes produce terpenoids (e.g., C_20_diterpenes) as defense. Often simpler structures but occasionally halogenated. They can contribute to the host sponge/coral’s chemistry. Still underexplored for diabetes and potential for sustainable production via fermentation.

**Table 2:** Marine vs. terrestrial terpenoids (key differences).

Aspect	Marine terpenoids	Terrestrial terpenoids
Structural diversity	Exceptionally high. Novel skeletons (e.g., halimane, sesterterpenes) with halogenation (Br, Cl) and extensive oxygenation are common (5, 25). Many are meroterpenoids (hybrid structures) or possess conjugated polyenes (e.g., carotenoids). Origin from both MVA and MEP pathways in some organisms adds variety (47)	Moderate. Largely derived from well known pathways (MVA in higher plants). Typically lack halogens; structures often limited to common motifs (steroids, triterpene saponins, etc.). Fewer polyhalogenated or highly conjugated terpenoids. Exceptions exist (e.g., some plant resins), but diversity is lower (47).
Potency & bioactivity	Often more potent antioxidative and antidiabetic effects. Many marine terpenoids act at micromolar or sub-micromolar concentrations in assays, showing multi-target activity (e.g., radical scavenging and enzyme inhibition) (5). For example, marine plastoquinones from algae inhibit PTP1B at 5–14 μM and α glucosidase at ~40–100 μM (46). Marine terpenoids frequently exhibit dual antioxidant and anti-inflammatory effects, improving glucose control and preventing complications in models (8, 17)	Often effective but can require higher doses or work best in combinations. Many plant terpenoids (e.g., monoterpenes in essential oils) have antioxidant activity but relatively modest antidiabetic efficacy unless used in high concentration or alongside other phytochemicals (5). Some plant triterpenes (e.g., ginsenosides) are potent but usually target one primary pathway (like PPARγ activation) and may not inherently have broad ROS-scavenging ability. Overall, marine terpenoids are noted to outperform terrestrial analogues in antioxidant capacity in comparable assays (5)
Pharmacokinetics (PK)	Often challenging PK profiles due to larger molecular weight and lipophilicity (35). Many marine terpenoids have poor water solubility and thus low oral absorption [e.g., astaxanthin oral bioavailability only ~10–50% (35)]. They may be rapidly metabolized or eliminated if not formulated properly (35). Specialized delivery systems (lipid nanoparticles, cyclodextrin inclusion, etc.) can improve bioavailability (35)	Highly variable. Small plant terpenes (monoterpenoids like menthol) are often lipophilic but small enough to have decent absorption; however, they may be cleared quickly. Larger plant terpenoids (triterpenoid saponins like glycyrrhizin or ginsenosides) suffer from poor absorption and extensive first-pass metabolism, similar to marine counterparts. Some plant-derived terpenoid drugs (e.g., taxol, an anticancer diterpenoid) required formulation strategies for delivery, which underscores that both marine and terrestrial terpenoids face PK hurdles when used as therapeutics

Marine terpenoids differ notably from plant derived (terrestrial) terpenoids in structure, potency, and often pharmacokinetics, as summarized in Table 2.

The above comparison emphasizes that marine terpenoids bring unique structural chemistries that can translate into higher pharmacological potency but also present formulation challenges. Their often-poor intrinsic bioavailability is a shared issue with many terrestrial natural products, but modern techniques (nano-formulations, prodrug approaches) are being applied to marine compounds to overcome this (35). Importantly, the distinct biosynthetic and ecological context of marine terpenoids gives them a competitive edge in multi-faceted action against diabetes, as a disease with complex, multi system pathology. Harnessing these differences effectively could enrich the current repertoire of antidiabetic agents with more potent and multitarget drugs. In light of these insights, further comprehensive studies are needed to map out how marine vs. terrestrial terpenoids differ in mechanisms at the molecular level. Such understanding will guide us in selecting or engineering compounds (including hybrids) that maximize therapeutic effects while minimizing drawbacks.

## 4 Antioxidant mechanisms of marine terpenoids

Marine terpenoids employ different strategies for antioxidant activity that are very important for the therapeutic implications they have in ameliorating oxidative stress. Their potential for radical scavenging, cellular antioxidant defense modulation, redox-sensitive signal regulation, and metal chelation provides a diverse basis for tissue and metabolic protection, thus supporting the role of marine terpenoids as innovative therapeutic interventions (see Figure 1).

### 4.1 Direct radical scavenging activities

The radical scavenging activity by marine terpenoids demonstrates their ability to scavenge free radicals to prevent oxidative stress in chemical-based assays of DPPH and ROS measurement. Puupehenone, a marine sponge sesquiterpenequinone, presents high radical scavenging activity with IC50 6.9 μM, which is even better than some marine or terrestrial terpene compounds with antioxidant effect, showing the potential ability of marine terpenoid compounds in treating oxidative stress, leading to diabetes and other metabolic diseases (36).

The radical scavenging ability of marine terpenoids directly reflects their structure-activity relationship. For example, phenol and ortho-hydroxyl groups may enhance the radical scavenging ability of marine terpenoid compounds such as compound 14 with low IC50 9.9 μM (36). These evidences suggest that the chemical structure of marine terpenoid compounds is a crucial consideration for its application as antioxidant therapeutics, especially when designing and applying novel marine terpenoid compounds. The stability and durability of marine terpenoids in eliminating free radicals in diabetic tissue also correlate to their efficacy in alleviating tissue damage in diabetes.

Marine terpenoid as direct antioxidant compounds for diabetes can be very helpful to neutralize all kinds of free radicals and prevent the further destruction of the cell membrane and lipid peroxidation. Furthermore, marine terpenoids are more functional in protecting against glucose-related damage, particularly in diabetes where excessive ROS causes pathological damages to multiple parts of the body (36).

DFT methods, with the help of computation, could show the real radical scavenging potential of marine terpenoids with a mechanistic approach. For example, the radical scavenging mechanism of SHQA by DFT reveals the antioxidant efficiency with a low BDE for O-H in SHQA structure, narrow HOMO-LUMO gap, and easy electron donating for radical quenching (37). SHQA also exhibited great activity as an antioxidant by low BDE and thus efficiently quenched free radicals in cellular environments.

In addition, the more chemical softness and polarizability properties also support the enhanced efficiency in the radical scavenging ability of marine terpenoid compounds, such as those in marine diatoms, which is able to cause a rapid charge transfer with free radicals compared to its terrestrial equivalent (37). This can be very important because long-term elevated ROS exposure to the body causes great damage to chronic diseases, such as diabetes. In general, it is assumed that marine terpenoid compounds evolved in the water to have these special properties, thereby promoting the function of quenching free radicals.

The role of some marine terpenoid compounds as direct antioxidants can be observed in cell models. Compound 11 as a cellular antioxidant enhances enzyme activity in the cells, scavenges free radicals, and induces other activities, providing a cellular defense system to eliminate free radicals in the cell, reducing oxidative damage (36). Marine terpenoids can exhibit various roles and perform different mechanisms in oxidative stress alleviation.

In addition, ROS levels in the cells directly relate to cell survival through mitochondria because excess ROS in oxidative stress may disturb mitochondrial membrane potential, resulting in cell death. In the diabetic environment, damage in mitochondria results in a pathological change to the cellular process, and marine terpenoids were able to prevent this change through direct antioxidant effects (36).

Marine biota also has complex chemical profiles that can be very different from those on the land, which have developed their defense strategies in different environments for a long evolutionary process, particularly those marine plants with a longer lifespan (38). For example, compared to their terrestrial relatives, marine algae have superior activities in radical-neutralizing capacity due to their complex chemical diversity, structural uniqueness, and biochemical modifications, which may contain various functional groups for radical scavenging activity. This may be due to the aquatic environment influencing the structures and properties of molecules present in aquatic organisms. Specifically, these characteristics, such as the incorporation of halogens, impart additional pharmacological advantages on these marine compounds in diabetic management.

Due to the radical scavenging mechanism, it is possible that one marine terpenoid compound can directly affect multiple pathways in oxidative stress in diabetes and protect multiple components in the cells, thereby serving as an antioxidant-based treatment for chronic disease. The natural availability in multiple biological sources could have advantages compared to other pharmaceuticals with limited bio-functionality (38). In this respect, more effort is needed to explore and develop this potential pharmaceutical from the ocean.

A recent overview in marine pharmacology provides a thorough literature review in pharmaceutical and medicinal applications of marine terpenoid-based compounds with direct antioxidant activities (39). While some other recent pharmaceutical discoveries are only limited to certain therapeutic activities, the bioactive marine organisms can also be rich sources for antioxidant and enzyme modulation of diabetes for both prophylactic and therapeutic treatment. For this aspect, the radical scavenging effects in response to the multiple metabolic functions would need to be better explored for clinical applications.

The effects of some marine-derived compounds have also shown promising potential in inhibition of carbohydrate-digesting enzymes to alleviate hyperglycemia. The bromophenols in marine biota of *Rhodomela confervoides* and *Symphyocladia latiuscula* present potential inhibitory activities against a-amylase and a-glucosidase. For example, bis-(2,3,6-tribromo-4,5-dihydroxybenzyl) ether, purified from *Rhodomela confervoides*, inhibited the function of a-amylase *in vitro* with IC50 0.03 ± 0.001 μM (40).

Future efforts should focus on enhancing the utilization of marine terpenoid-based pharmaceuticals by further exploring the multiple functions of marine terpenoids in metabolism, redox activities, and other relevant pathways. Through integration of these properties, they may be developed as effective and strategic interventions for patients of oxidative-related diseases.

### 4.2 Modulation of cellular antioxidant defenses (e.g., SOD, CAT, GPx, GSH)

Marine terpenoids demonstrate the ability to strengthen cellular antioxidant defense systems in diabetes by modulating the activity of antioxidant enzymes like superoxide dismutase (SOD), catalase (CAT), and glutathione peroxidase (GPx) and by increasing the level of glutathione (GSH), which has antioxidant properties. In order to treat diabetes and/or prevent diabetes-related health problems, numerous marine-derived products can be used to reinstate redox balance and avert oxidative damage in diabetic tissues. By improving the activity of antioxidant enzymes such as SOD and GPx, terpenoid-rich marine extracts significantly lower the blood glucose level in diabetic rats. Moreover, these marine extracts have a significant impact on reducing oxidative stress (10, 14).

Fucosterol and squalene are terpenoids derived from marine algae. Fucosterol has been reported to suppress pro-inflammatory biomarkers and enhances SOD and CAT activities via regulation of the PI3K/Akt/NF-κB signaling pathway in the liver. Hence, this terpenoid plays a role in preventing diabetes. Besides fucosterol, squalene, another terpene isolated from marine algae, plays a key role in scavenge ROS and activates antioxidant enzyme activity to prevent hyperglycemia-induced oxidative damage (14). Further studies can be conducted to observe whether this finding can be reproducible in different models, dosages, and time frames, considering that various variables such as glycemic variability, insulin deficiency, and differences in metabolic mechanisms could affect the results obtained.

Marine terpenoids play a crucial role in increasing intracellular GSH, an important non-enzymatic antioxidant, by scavenging ROS and converting other antioxidants back into their active forms. Low GSH levels are commonly observed in diabetic patients. By inducing antioxidant enzymes SOD and GPx and increasing intracellular GSH levels, marine peptides and crude extracts, containing terpenoids, can significantly combat oxidative stress during diabetes (15). A few marine peptides, for instance, exhibit remarkable *in vitro* and *in vivo* antioxidant capabilities against several oxidative stress-related diseases such as diabetes. These peptides have the ability to increase the levels of GPx, SOD, and GSH in cells and mice (10). This increased intracellular GSH contributes to a reductive environment, protecting β-cells against damage. Additional study may be beneficial in further confirming and elaborating the potential mechanisms of marine-derived products in improving cellular redox balance.

Apart from improving antioxidant activity, marine terpenoids are beneficial in preventing and relieving organ-specific damage. Oxidative stress is regarded as an important driver of tissue damage and thus leads to the pathogenesis of diabetic diseases. Some marine terpenoids are capable of protecting and enhancing the activity of vital organs in diabetics, such as the kidneys, liver, and vasculature. They are also helpful in regulating the redox state of tissues, preventing lipid peroxidation, and suppressing DNA damage to alleviate the pathogenesis of diabetic complications (10, 13). However, there are still many uncertainties regarding the safety and efficacy of marine-derived products, particularly for their prolonged uses, their accumulation in tissues, and the subsequent formation of bioactive metabolites.

Several studies on the effectiveness of marine terpenoids on redox balance indicate that they can trigger the Nrf2/HO-1 pathway, a redox-sensitive signaling pathway that is activated by various external stress factors. This results in increased production of detoxifying enzymes and antioxidant proteins (10, 16). The activation of Nrf2 pathway induces the accumulation of Nrf2 protein in the nucleus, enhances transcription of HO-1 mRNA, and elevates HO-1 expression. The production of HO-1 helps maintain cellular homeostasis through the regulation of oxidative stress by producing several important downstream metabolites. However, the specific Nrf2-inducing fragments, as well as the structure-activity relationship, in the marine-derived products need further assessment and examination.

One of the key factors of oxidative stress during diabetes is ROS overproduction as a result of the activity of NOX enzymes. NOX2 and NOX4 are important pro-oxidant enzymes in the production of ROS. Interestingly, several marine terpenoids have been found to suppress the activity of these enzymes, in turn restoring redox balance in cells (14, 16, 33). On the contrary, NOX isoforms, which are members of NADPH oxidase enzyme family, are found to promote and accelerate the effects of ROS. More research is required in order to discover potential antioxidants that may reduce or alleviate NOX isoforms in diabetic patients.

Bioinformatics analysis combined with vitro assays have exhibited that the potential therapeutic targets of marine terpenoids for preserving β-cell viability and improving insulin secretion can be attained by modulating β-cell function and endogenous antioxidant defenses. Hence, marine terpenoids have the potential to promote better glycemic control and prevent β-cell dysfunctions during diabetes by stimulating the endogenous antioxidant defense mechanisms. This can also decrease the pathogenesis of diabetes and its complications (13). These findings suggest that further research and clinical investigations are warranted to validate these initial outcomes of the effect of marine-derived products on antioxidant activity and β-cell preservation during diabetes.

In summary, marine terpenoids have a wide range of antioxidant effects on biological systems, from activating enzyme systems to elevating GSH and stimulating redox-sensitive pathways. The protective benefits of marine terpenoids for people with diabetes have also been exhibited in preclinical studies. However, it is necessary to have in-depth analyses of their mechanisms of action and long-term effects so that these bioactive compounds can be developed into valuable therapeutic approaches to prevent and treat diabetes and its complications.

### 4.3 Regulation of redox-sensitive signaling pathways (e.g., Nrf2/ARE, NF-κB)

Marine terpenoids significantly modulate redox sensitive signaling pathways, especially in the management of oxidative stress and diabetes. One of the most crucial pathways, the Nrf2/ARE pathway, plays a vital role in combating oxidative damage by enhancing the expression of key antioxidant enzymes. Stimulation with marine terpenoids leads to the release of the nuclear factor erythroid 2-related factor 2 (Nrf2) from the cytoplasm, allowing its translocation to the nucleus and its binding to antioxidant response elements (AREs). This in turn promotes the transcription of HO-1, SOD, and CAT. In various diabetes models, marine terpenoids, such as phlorotannins and fucoidan, have been shown to increase antioxidant enzyme activity in both *in vitro* and *in vivo* conditions. Their treatment decreases ROS accumulation, inflammation, and apoptosis to alleviate tissue injuries, as well as protects the organs from diabetic complications (10, 14). HO-1, in particular, also exerts inhibitory effects on inflammation and apoptosis that contribute to organ protection against oxidative stress and diabetes. The Nrf2/ARE pathway has an important role in maintaining cellular redox balance, making marine terpenoids vital in oxidative stress-associated diabetes management.

A noteworthy contribution of marine terpenoids is that they can effectively inhibit the NF-κB signaling pathway. The NF-κB pathway plays a major role in modulating the inflammatory response, which, in the context of diabetes, is commonly activated because of an increase in ROS. Chronic inflammation can contribute to poor insulin resistance and reduced β-cell function, resulting from the elevated levels of pro-inflammatory cytokines (IL-6 and TNF-α) and enzyme COX-2. Marine terpenoids, such as fucosterol and squalene, effectively suppress the activation of NF-κB. Inhibition of this pathway consequently reduces the production of pro-inflammatory cytokines and alleviates the long-lasting inflammation associated with diabetes (13, 14). Preclinical studies on murine models show that the administration of marine terpenoids can help reduce not only systemic but also organ-specific inflammation (33).

Nrf2 and NF-κB activation are mediated by PI3K/Akt and ERK signaling kinases, which are activated in response to marine terpenoids to enhance Nrf2 translocation and HO-1 expression. Meanwhile, activation of these signaling pathways inhibited NF-κB activation, as it can prevent inflammation by inhibiting the transcription of pro-inflammatory genes. Therefore, activation of these signaling kinases by marine terpenoids plays an important role in the prevention and management of diabetes and its complications by increasing antioxidant defenses, mitigating inflammation, and reducing oxidative damage (14). The mechanisms involved and their activation of Nrf2 and NF-κB highlight the effectiveness of marine terpenoids in improving diabetic health and lowering the severity of the disease, as well as limiting oxidative damage and poor inflammation status.

Preclinical studies show that marine terpenoids improve diabetic health and reduce disease severity. Administration of these marine compounds has been shown to improve lipid profiles, reduce oxidative damage to the tissues, and prevent diabetic complications, such as renal and hepatic injury, which has been shown *in vivo* (10, 17). Furthermore, marine terpenoids possess antioxidant activity by inducing Nrf2 and inhibiting the NF-κB signaling pathways to manage oxidative damage and regulate inflammation. For example, marine terpenoids prevent vascular and endothelial injury, which prevents atherosclerosis, a diabetic complication (17). Overall, the potential of marine terpenoids in oxidative stress-associated diabetes prevention and management is apparent in several studies, but further research and clinical trials are needed to evaluate its true impact.

Despite these findings, there are some research gaps that need to be addressed. Current data mainly comes from animal models, highlighting the lack of data on the long-term effects of the pathway in human populations and how its long-term modulation with marine terpenoids affects overall health (10). Some marine terpenoids are not bioavailable, so more research is needed on the delivery and effectiveness of these compounds. The variability of the dose–response and mechanism effects of some marine terpenoids in different diabetic models suggests the importance of controlled trials. The long-term exposure to marine terpenoids needs further clarification concerning potential off-target effects, which emphasizes the importance of further studies that characterize the long-term safety effects and clinical efficacy in diabetes prevention and management. Furthermore, well-controlled randomized trials need to be conducted to evaluate clinical safety and efficacy of treatment interventions.

### 4.4 Metal chelation and inhibition of pro-oxidant enzymes

Marine terpenoids exhibit the ability to bind pro-oxidant metals and inhibit pro-oxidant enzymes to reduce oxidative stress. The binding of pro-oxidant transitional metals like iron (Fe^2+^/Fe^3+^) and copper (Cu^+^/Cu^2+^) by marine terpenoids is a major mechanism of antioxidant action. These metals are vital mediators of Fenton and Haber-Weiss reactions, responsible for hydroxyl radicals that exacerbate oxidative stress in diabetics. By directly chelating these redox-active metals, terpenoids help avert the progression of lipid peroxidation and oxidative damage. The hydroxyl group, the presence of conjugated systems, and sometimes the halogenated sites in marine terpenoids play a role in metal chelation. This is the distinguishing structural feature that may contribute to the higher metal-binding ability and improved antioxidant function, compared to their terrestrial analogs. This would potentially explain the better antioxidant activity of marine-derived terpenoids in preclinical diabetes models (13). However, the variability of the chelation ability among the marine terpenoids should be studied in order to estimate their contribution to the chelation process. Also, the stability of chelation under diabetic conditions should be demonstrated.

Another way by which marine terpenoids exert their antioxidant effects in diabetes management is by inhibiting pro-oxidant enzymes, NOX, myeloperoxidase, and xanthine oxidase. It has been observed that NOX is an NADPH-dependent pro-oxidant enzyme that produces reactive oxygen species in diabetics. Marine terpenoid extracts attenuate NOX-dependent superoxide generation and myeloperoxidase activity in diabetic tissues, diminishing ROS and lipid peroxidation. This has a beneficial effect on pancreatic β-cell viability (14). In addition, xanthine oxidase, which converts hypoxanthine and xanthine to uric acid, is also inhibited. Thus, marine terpenoids can reduce ROS production and increase antioxidant status in diabetes. However, it is necessary to check these effects across different experimental models.

Moreover, phlorotannins from brown algae are notable terpenoids that exhibit both metal chelation and inhibition of pro-oxidant enzymes, contributing to their potent antioxidative capability. They have been reported to inhibit NOX and xanthine oxidase, thus exerting antioxidative effects in preclinical models (18). Additionally, squalene isolated from brown algae is a very good quencher of singlet oxygen and activator of antioxidant enzymes (14).

Also, *Ulva reticulata*, a green seaweed rich in terpenoids, can control blood glucose and also inhibit oxidative enzymes in experimental diabetes (19). It has been observed that certain terpenoid compounds bind directly to the active sites or metal ions of oxidative enzymes, reducing the overall catalytic activity through a variety of mechanisms such as conformational changes and blockage of the active site or cofactor site. Thus, *Ulva reticulata* suppresses the production of ROS and lowers the rate of glucose metabolism under pathological conditions. Nevertheless, it is essential to determine the precise functional structural attributes of marine-derived terpenoids from this seaweed that have the inhibitory effect against the selected oxidative enzymes.

Generally, marine terpenoids exhibit superior metal-chelating and pro-oxidant enzyme-inhibitory capabilities due to their unique structural features. Structural characteristics, such as ortho-hydroxyls, conjugated polyenes, and halogens, are more often present in marine-derived terpenoids compared to their terrestrial counterparts, leading to increased capacity to inactivate transition metals and influence enzyme activity (13, 39). As marine organisms have to face stressful situations such as high salinity, lower oxygen levels, or fierce competition for niche occupation, it is reasonable that their biochemical systems have developed strategies, like those in terpenoids, for survival.

**Figure 3:**
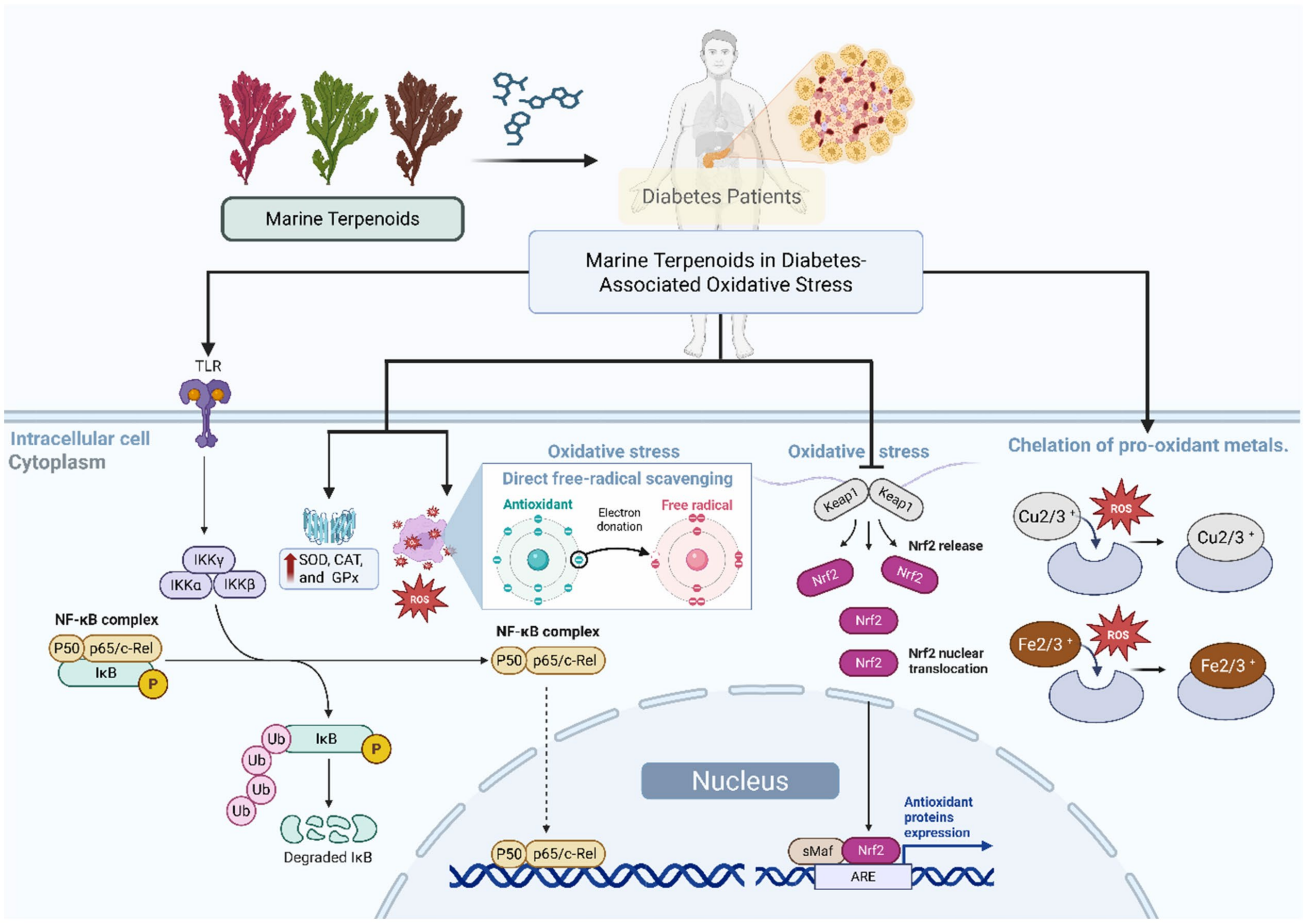
Mechanisms of antioxidant and antidiabetic action of marine terpenoids. Proposed mechanisms by which marine terpenoids ameliorate diabetes-associated oxidative stress: (1) direct free-radical scavenging, (2) enhancement of antioxidant enzyme activity (SOD, CAT, GPx), (3) regulation of redox-sensitive signaling pathways (Nrf2/ARE activation, NF-κB inhibition), and (4) chelation of pro-oxidant metals.

Despite the promising effects of marine terpenoids in controlling oxidative stress, additional studies are required to clarify their mode of action (Figure 3). Specifically, more research is needed to determine the precise interaction of these terpenoids with metal ions and enzyme targets to better understand their antioxidative and antidiabetic activities (13, 18). Although the majority of the studies reported in the literature were performed *in vitro*, most of them are not consistent with the physiological conditions observed *in vivo*. Further studies are needed to evaluate their efficacy as antioxidant agents *in vivo* through properly designed controlled experiments. Also, studies are needed to fully comprehend the bioavailability, metabolism, and possible toxicity of marine terpenoids in order to confirm their suitability for clinical application. Hence, additional studies of marine terpenoids in well-controlled experimental models of diabetes through molecular modeling and bioassays should lead to a better understanding of the application of marine-derived terpenoids in diabetes.

## 5 Preclinical evidence: marine terpenoids in diabetes models

The preclinical studies explore the promising potential of marine terpenoids in diabetes management by examining their protective effects at cellular, tissue, and organ levels. Focusing on *in vitro* and *in vivo* models, this section highlights how these compounds improve glycemic control, reduce oxidative stress, and shield against diabetic complications. Situated within the broader context of marine bioactives’ therapeutic capabilities, these findings underscore the translational relevance and future prospects of marine-derived compounds in combating diabetes (see Figure 2).

### 5.1 *In vitro* studies (cellular protection, ROS assays, β-cell preservation)

Marine terpenoids are useful compounds of *in vitro* studies because of their cytoprotective, antioxidant, and anti-inflammatory activity that may have a crucial role in diabetes. Their protection of pancreatic β-cells from oxidative damage is of significant interest as a result. Under hyperglycemic conditions, there is an overproduction of intracellular reactive oxygen species (ROS), which stimulates apoptotic and necrotic pathways, leading to β-cell damage. Marine terpenoids such as fucosterol, a triterpene isolated from marine algae, can protect β-cells by activating the antioxidant enzymes glutathione peroxidase (GPx) and superoxide dismutase (SOD) (14). This mechanism helps to decrease ROS levels and improves both mitochondrial and DNA integrity (10). The ability of marine terpenoids to preserve β-cell viability is necessary as loss of the pancreatic β-cell population is a critical step in the etiology of diabetes. More research must be done to elucidate any limitations of the treatment, for example, whether the effects vary among β-cell lines, and to determine whether the beneficial mechanisms are regulated through intracellular signaling pathways.

Marine terpenoids can also scavenge for ROS *in vitro*. This is evident in the marine algal and animal-derived terpenoid squalene and algal phlorotannin, which scavenge for ROS and inhibit lipid peroxidation. Under conditions of hyperglycemia-induced stress in *in vitro* models, these marine terpenoids show marked improvement in cell redox status due to increased activity of antioxidant enzymes, including SOD, catalase (CAT), and GPx (10). The resulting improved redox environment may preserve cells from oxidative damage and ensure cell integrity. This is an essential attribute of marine terpenoids as hepatocyte and myocyte cultures are prone to oxidative damage in diabetes. Compared to artificial antioxidants, marine terpenoids may sustain beneficial redox effects via modulation of cellular antioxidant defense programming rather than transient ROS scavenging. The mechanism in which marine terpenoids stimulate the upregulation of these enzymatic pathways is still not well understood, as well as the structural features of terpenoids involved.

Terpenoids isolated from marine invertebrates possess not only antioxidant effects but anti-inflammatory effects, important in ameliorating diabetes-induced endothelial and tissue damage. *In vitro* studies have demonstrated the ability of marine terpenoids to suppress ROS and simultaneously suppress the release of pro-inflammatory cytokines, such as interleukin-1β (IL-1β) and tumor necrosis factor-α (TNF-α) (33). As both oxidative stress and inflammation act in tandem to bring about endothelial damage and inflammation, it is paramount to control their production to mitigate pathogenesis in diabetes. Marine terpenoids also reduce the gene and protein levels of pro-inflammatory cytokines, alleviating their contribution to the positive feedback loop that propagates oxidative damage and inflammation. Therefore, marine invertebrate-derived terpenoids can also protect endothelial and immune cells from damage due to inflammation. The validity of these findings and effects of the compounds on other pathological processes of diabetes need further investigation as most cell cultures investigated *in vitro* were isolated from healthy or inflammatory disease-free organisms.

Extracts rich in marine terpenoids were also found to enhance insulin-mediated glucose uptake in adipocytes and myocytes. Studies measured *in vitro* glucose uptake in cells by performing a glucose transport assay using two muscle cell lines and one adipocyte cell line after treatment with the extracts. The results indicated a significant dose-dependent increase in the cell’s glucose uptake due to an enhancement of glucose transporter type 4 (GLUT-4) translocation, the main glucose transporter involved in peripheral glucose uptake. Other investigations have shown that these marine terpenoids simultaneously promote antioxidant pathways (20). The ability to activate both insulin-stimulated glucose uptake and the body’s own antioxidants is a significant attribute as oxidative stress directly contributes to insulin resistance. This suggests that marine terpenoid treatment *in vitro* may have combined benefits for diabetics. It is still not clear, however, what are the steps that link these two mechanisms and what exact roles or pathways are utilized by the marine terpenoids. Thus, more studies are needed to determine whether there are any common intermediaries that coordinate both mechanisms or if they work synergistically or additively.

Some marine terpenoids are also capable of regulating blood glucose levels. Many essential oils of terpenoids found from marine macroalgae and microbes, specifically oils composed of hydroxylated marine terpenoids, are shown to significantly inhibit pancreatic α-amylase, an enzyme that breaks down complex carbohydrates in the intestine and pancreas into monosaccharides, increasing absorption in the blood and the postprandial blood glucose level. For example, essential oils from several species in Eucalyptus, along with species from *Juniperus phoenicea*, possess this inhibitory effect (41). Marine terpenoids also control postprandial blood glucose levels by lowering ROS-induced lipid peroxidation. The addition of terpenes and terpenoid-rich fractions of essential oils decreases lipid peroxidation levels, as measured by the β-carotene-linoleic acid system, in a dose-dependent manner. The benefits shown in these examples support the importance of some terpenoids, and therefore their possible applications as novel antidiabetic agents. More research needs to be carried out, however, to examine the bioavailability and selectivity of these compounds to regulate blood glucose levels in human physiological settings.

Nrf2/HO-1 activation is one of the mechanisms that marine terpenoids use to achieve antioxidant effects. Activation of this pathway activates the transcription of the genes of antioxidant enzymes, and activation of the pathway with marine terpenoids has significantly protected pancreatic β-cell architecture under oxidative stress conditions *in vitro* (10). Overall, activation of Nrf2/HO-1 by marine terpenoids represents a plausible mechanism that directly mediates beneficial effects to ameliorate oxidative stress *in vitro*.

In conclusion, marine terpenoids have great potential as therapeutic agents because of their abilities to reduce oxidative stress, maintain healthy cell viability, and enhance glucose transport, according to *in vitro* studies. The range of effects achieved by marine terpenoids may even extend beyond oxidative stress as *in vitro* experiments have demonstrated that these compounds simultaneously attenuate inflammation and activate intracellular antioxidants by stimulating the Nrf2/HO-1 pathway. In addition to the limitations of the present study, further studies are still needed to validate these findings *in vivo*.

### 5.2 *In vivo* animal studies (glycemic control, oxidative markers, organ protection)

*In vivo* studies confirm that marine terpenoids demonstrate potential in the management of diabetes by improving glucose control, oxidative stress, and organ damage. Evidence suggests that marine terpenoids, like *Padina pavonia*, ameliorate hyperglycemia in diabetes. They improve both fasting and postprandial blood glucose levels and are associated with an increase in the activity of hepatic hexokinase, which promotes glycolysis. Moreover, they decrease the activity of gluconeogenic enzymes (glucose-6-phosphatase and fructose-1,6-bisphosphatase), leading to reduced endogenous glucose production (42). PPARγ expression in the liver increased when treated with marine terpenoids. All these changes may indicate improved glucose homeostasis via enhanced insulin sensitivity and the metabolism of lipids. They lower total cholesterol and triglyceride levels, which are frequently raised in dyslipidemia associated with type 2 diabetes. Despite their benefits, it still remains to be investigated whether the same results can be achieved in other diabetic models, which represent a broader, heterogeneous population.

Marine invertebrate-derived terpenoids are capable of mitigating oxidative stress by reducing malondialdehyde and significantly increasing the activity of antioxidant enzymes such as superoxide dismutase, catalase, and glutathione peroxidase. Simultaneously, there is a decrease in pro-inflammatory cytokines, like IL-6 and TNF-α. By reducing oxidative stress and inflammation, marine invertebrate-derived terpenoids prevent the activation of apoptotic pathways, leading to reduced markers of hepatic and renal injury (33). Histological analysis reveals reduced infiltration of fat droplets in liver and kidney cells and the amelioration of the tissue structure of these organs, suggesting the prevention of damage to them. This evidence suggests that marine invertebrate-derived terpenoids, by reducing both oxidative and inflammatory stress, achieve what many current antidiabetic medications do not. Nevertheless, there is a need for extended-duration studies for a full understanding of their effect on preventing organ damage during diabetes.

High-fat diet-induced diabetic models supplemented with marine-derived terpenoids show improved HOMA-IR, which signifies enhanced glucose homeostasis. In general, marine terpenoids were shown to reduce postprandial blood glucose (21, 22). The suggested mechanism is the inhibition of carbohydrate-digesting enzymes (α-amylase and α-glucosidase), which results in slower glucose absorption. Moreover, marine terpenoids can protect against oxidative stress and inflammation by reducing the activation of NF-κB. This translates to lower cytokine and malondialdehyde levels in the vasculature, liver, and kidneys, consequently ameliorating vascular and renal injury markers. Histological studies show that this lowers the level of fibrosis and inflammation. Therefore, the vascular-and renal-protecting effects of marine terpenoids may not only be associated with their ability to ameliorate blood glucose and glucose metabolism but also with the reduction of oxidative stress and inflammatory cytokine production. However, further studies are necessary to determine the optimal dose, as some studies show no ameliorating effect. Furthermore, treatment duration and bioavailability are of paramount importance for achieving their therapeutic effects.

According to various experimental studies, marine-derived terpenoids, such as dehydroeburicoic acids and hydroxylated terpenoid-enriched essential oils, are shown to be more beneficial than standard antidiabetic drugs like metformin and fenofibrate in terms of glycemic and antioxidant effects (41). Some of them, such as hydroxylated terpenoid-enriched essential oils, reduced blood glucose more significantly than metformin. These studies support the superior ability of marine terpenoids to reduce blood glucose and improve antioxidant markers. The inhibition of carbohydrate-digesting enzymes (α-amylase and α-glucosidase) by marine terpenoids helps improve the postprandial glycemic index by reducing the rate of glucose absorption. This also means they address the source of the glucose peak, and not only the metabolic results of it. Therefore, marine terpenoids are superior to conventional drugs since they possess both glucose-lowering and antioxidant properties. Yet, more head-to-head comparisons with standard drugs are required to support that statement because, in general, comparisons between studies are limited.

Marine terpenoids reduce indicators of inflammation, fibrosis, and oxidative stress in diabetic nephropathy. More specifically, the treatment of diabetes-induced nephropathy with fucoidan and phlorotannin results in the downregulation of pro-fibrotic marker TGF-β, which is heavily involved in the fibrosis progression of the kidney (23, 24). Overall, the evidence from *in vivo* studies supports the hypothesis that marine terpenoids mitigate renal damage, at least in mice models of diabetes, because they reduce markers of fibrosis and renal tissue damage. Yet, whether they ameliorate kidney function in other animal models of diabetes, as well as in different stages of diabetes, is yet to be investigated. Moreover, since these substances may have undesirable side effects, studies are also required to assess whether their anti-fibrotic and anti-inflammatory effects can be confined to renal tissue or if they affect other organs as well.

To conclude, this chapter discussed the effects of marine-derived terpenoids *in vivo*. Although there are still many variables left to investigate, the majority of *in vivo* studies support the potential of marine terpenoids as an adjunct or replacement to currently used antidiabetic drugs. This is because the discussed studies indicate their hypoglycemic and antioxidant properties may provide vascular and renal protection by decreasing inflammatory cytokine and malondialdehyde expression. In short, *in vivo* studies indicate that marine-derived terpenoids may have great therapeutic potential. Therefore, in the future, scientists must address the gaps in knowledge and lack of studies for these substances to be tested in humans.

### 5.3 Highlighted marine terpenoids with strong antidiabetic-antioxidant activity (e.g., sargaquinoic acid, fucosterol, halimane diterpenoids, etc.)

Marine terpenoids, or terpenoids of marine origin, have demonstrated promising antidiabetic and antioxidant efficacy. For example, the marine terpene sargaquinoic acid has displayed dual functionality as a free radical scavenger and hypoglycemic effect. Sargaquinoic acid, isolated from the brown algae species, has strong free radical scavenging properties. This may be caused by the quinone structure. Its highly conjugated nature is crucial for its electron donation capabilities. In enzymatic inhibition studies, sargaquinoic acid has been found to inhibit α-glucosidase with great potency. This hypoglycemic effect has been found to be dose-dependent (33). More studies are required to ascertain if sargaquinoic acid can be a promising candidate in diabetes prevention. Structural analogs may improve target specificity as well as stability for effective development.

Another example of a marine terpene showing potent antidiabetic and antioxidant effects is fucosterol. Fucosterol is a sterol derived from various marine algae, such as *Undaria pinnatifida* and *Ecklonia stolonifera*. It exerts hypoglycemic and antioxidant functions. The molecular mechanisms involved are aldose reductase inhibition, α-glucosidase inhibition, as well as upregulated antioxidant enzymes like superoxide dismutase (SOD) and catalase (CAT) (14). It has been found to suppress interleukin-6 (IL-6) and cyclooxygenase-2 (COX-2) via modulation of PI3K/Akt/NF-κB signaling pathways (26). The overall mechanisms involved are improving insulin sensitivity, decreasing serum glucose levels, inhibiting carbohydrate digestion, and preventing the production of oxidants as well as pro-inflammatory factors. More in-depth exploration into the bioavailability, long-term safety of the compound, as well as extensive pharmacokinetic evaluations may be necessary.

Sesquiterpenes and triterpenoids isolated from *Padina pavonia* have proven to have great antioxidant and antidiabetic effects by targeting nuclear receptors such as PPARγ. Upgrading the expression of PPARγ enhances lipid metabolism and insulin sensitivity in diabetes. These marine terpenoids also have been effective in reducing fasting glucose, glycosylated hemoglobin, as well as HOMA-IR. They have also enhanced glycolysis as well as glycogen content. It was proven that by enhancing hexokinase activities, and at the same time by inhibiting gluconeogenic enzymes such as glucose-6-phosphatase, these terpenoids from *Padina pavonia* corrected glucose metabolic dysfunctions. They also have potent antioxidant properties by enhancing serum as well as hepatic antioxidant enzyme activities (42). The exact molecular interactions between *Padina pavonia* terpenoids and PPARγ remain unclear and may be different depending on the experimental model.

Another set of potent marine terpenoids are phlorotannins like dieckol, phloroglucinol derivatives, and others extracted from marine brown algae like *Ecklonia cava*. They have proven to have very potent α-glucosidase inhibition activity with low IC50 values and radical-scavenging activities. The activity shown may be attributed to the abundance of hydroxyl groups in phlorotannin structures. Because of the potent free radical scavenging nature of phlorotannins, the suppression of lipid peroxidation has been discovered. Phlorotannins *in vivo* can improve glucose tolerance, insulin sensitivity, and ameliorate inflammation as well as oxidative stress-related tissue damage in diabetic conditions (27). These diverse modes of action may be beneficial in treating the onset of diabetes and its progression of complications. Standardization of phlorotannin-rich extracts is needed, however (26). Because the extraction method may vary across the literature, there is no clear standard compound. The stability of each phlorotannin-rich extract also needs to be verified for practical applications.

**Figure 4:**
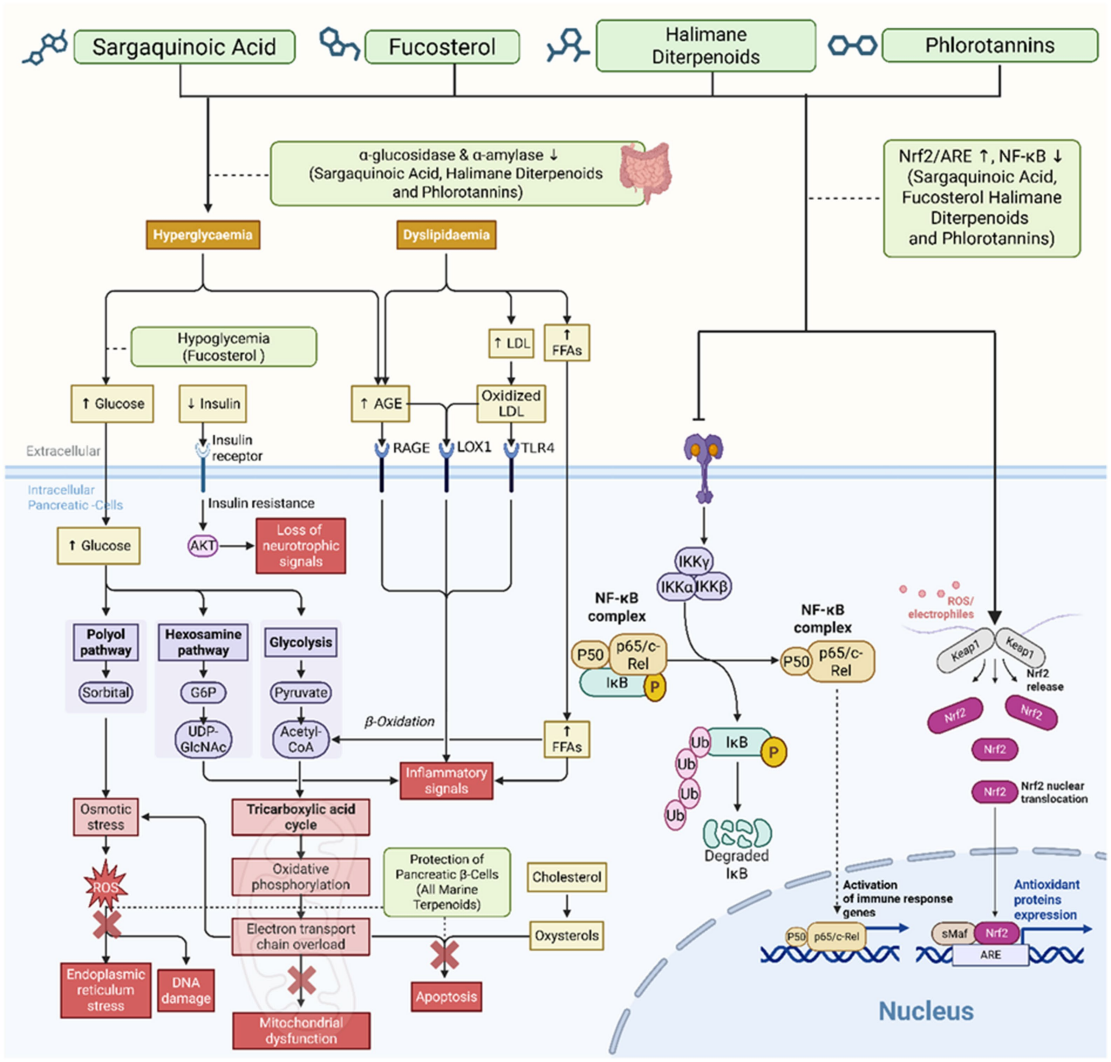
Highlighted marine terpenoids with dual antioxidant and antidiabetic effects. Structural representations and summarized bioactivities of key marine terpenoids—such as sargaquinoic acid, fucosterol, halimane diterpenoids, and phlorotannins—showing their main molecular targets and pharmacological actions in diabetes models.

Halimane diterpenoid and squalene, for example, have demonstrated many potential antidiabetic effects with antioxidant characteristics (Figure 4). These two terpenes have distinct chemical structures from each other but are both extracted from marine sources. Halimane diterpenoids exhibit prominent antioxidant effects due to the unique nature of the halimane skeleton, resulting in more potent pro-oxidant enzyme-binding characteristics as well as radical-scavenging abilities (33). Squalene also has great antioxidant ability by scavenging singlet oxygen species and by activating antioxidant enzymes such as SOD and CAT. More research and applications in this realm are still needed (14). Each compound of Halimane diterpenoids and squalene have multiple hydroxyl sites and unique conjugated systems. These structural properties are able to modulate redox, inflammatory, as well as metabolic pathways to exert beneficial effects against oxidative stress as well as other underlying mechanisms associated with T2DM. Because both these marine-sourced terpenoids are able to exert the antidiabetic effect by multiple modes of actions, these would be the ideal natural products for polypharmacology. More studies regarding the isolation techniques, structural characterization, as well as pharmacological parameters are warranted.

## 6 Potential mechanisms in diabetic complication prevention

Diabetes-related complications involve damage to vessels and nerves mediated by oxidative stress and inflammation. Marine terpenoids offer mechanisms to protect these tissues by impacting signaling pathways that maintain vessel and nerve integrity. Examination of these mechanisms highlights the potential of marine bioactive for addressing multiple features of diabetic pathology (see Figure 3).

### 6.1 Vascular protection (endothelial function, atherosclerosis)

Marine terpenoids and their antioxidant and anti-inflammatory effects make them great candidates for mitigating diabetic vascular complications. The Nrf2/HO-1 signaling pathway is able to activate the transcription of antioxidant defense cytoprotective genes, like heme oxygenase-1, SOD and CAT. The upregulated expression of these genes increases the antioxidant capacity by neutralizing reactive oxygen species (ROS), thus counteracting vascular endothelial cell damage (10).

The oxidative-scavenging action of these compounds reduces oxidative byproduct accumulation, therefore relieving diabetes-induced endothelial dysfunction. This phenomenon is largely due to decreased ROS accumulation and increased nitric oxide bioavailability. Nitric oxide is a potent vasodilator responsible for the preservation of vascular endothelial cell viability. Its reduced availability in diabetes results in endothelial dysfunction, as nitric oxide is also an important mediator of endothelial cell apoptosis (10).

Marine terpenoids were also shown to reduce certain markers of endothelial damage, like vascular cell adhesion molecules and endothelial cell permeability, indicating a role in protecting against vascular inflammation and atherosclerosis (42). These compounds can exert their beneficial effects against vascular inflammation by preventing endothelial cell disruption at its early stages due to their antioxidant activity. This is very important when taking into account the vascular remodeling that leads to arterial stiffening in diabetic individuals. These data suggest a promising role of marine terpenoids to improve both microvascular and macrovascular injuries in diabetes.

Another report supports that terpenoids-rich extracts from the marine algae *Padina pavonia* can induce vascular protection by improving insulin sensitivity and suppressing inflammation in addition to the activation of PPARγ (42). This leads to an increase of the glucose transporter GLUT-4 activity, thus improving glucose uptake. These effects are crucial to fight insulin resistance, a very common cause of endothelial dysfunction in diabetes. Taking into account all of these effects, marine terpenoids can have a comprehensive protection of blood vessels. In contrast, other studies demonstrated no association between marine terpenoids consumption and reduction of ROS levels, but there was improvement of aortic reactivity due to elevated nitric oxide, which promoted vascular relaxation. These conflicting data suggest the need to identify optimal treatment duration and concentrations of marine terpenoids in order to have efficient outcomes *in vivo*.

Moreover, the reduction of TNF-α and IL-6 levels by marine terpenoids further exerts an inhibitory effect on the onset of atherosclerosis (42). Those two cytokines promote the expression of vascular cell adhesion molecules, thus facilitating the recruitment of leukocytes to the endothelial cell layer and augmenting the inflammatory response in the blood vessels. This further demonstrates how these compounds interfere with the vicious circle established between oxidative stress and inflammation, ultimately inhibiting the onset of vascular damage in diabetes.

Marine terpenoids also suppress the activity of COX and LOX, two important enzymes that result in eicosanoids (prostaglandins and leukotrienes) production. These molecules induce the recruitment and activation of inflammatory cells to the vascular wall. Therefore, marine terpenoids are capable of preventing or reducing the severity and progression of diabetes-induced vascular inflammation and atherosclerosis (33). Marine terpenoids are also inhibitors of NF-κB activation, which is a major transcription factor of inflammatory cytokines, chemokines and adhesion molecules. Once activated, the expression of such molecules increases, therefore worsening oxidative stress and vascular inflammation. This self-perpetuating cycle contributes to the progression of endothelial dysfunction and atherosclerosis. Through NF-κB inhibition, marine terpenoids are able to disrupt this cycle by halting its further progression (10).

The lower levels of eicosanoids and reduced activation of the NF-κB pathway also resulted in the reduced progression of atherosclerosis in animal models and promoted vascular health. By preventing and/or reducing oxidative damage and vascular inflammation, marine terpenoids target key contributors to diabetes-induced vascular injury (33).

Also, they are inhibitors of LDL oxidation. Oxidatively modified LDL is a critical event in the onset of atherosclerosis. These LDL products have been shown to accelerate the monocyte and macrophage recruitment into the vascular walls, resulting in early atherogenesis. In response to LDL oxidative stress, these cells become highly inflamed and actively contribute to further ROS production. Furthermore, modified LDL is taken up by monocytes and macrophages through scavenger receptors, resulting in an increase of lipid storage within such cells, thereby favoring foam cell formation (41). This is important when taking into account that both monocytes and macrophages represent the major type of cells present in the atherosclerotic plaques. Marine terpenoids inhibit the oxidation of LDL through effective ROS scavenging, thus preventing lipid peroxidation. The reduced monocyte recruitment in the endothelium may have an important role in protecting against diabetes-induced vascular damage, since the inflammatory properties of this cell type lead to foam cell formation and accelerated plaque development in atherosclerosis.

Furthermore, the antioxidant effect of marine terpenoids can improve endothelial-dependent vasodilation (28). Increased nitric oxide production has been described in endothelial cells treated with marine terpenoids. Vasodilation caused by nitric oxide also favors the reduction of arterial stiffening. The vascular endothelial cells have decreased production of this factor due to increased oxidative stress and inflammation (28). Marine terpenoids have shown potent effects in vascular health in type 2 diabetes. However, the mechanisms, effects, and interactions of these effects can change the result of this activity and may not be effective or even change their function. Therefore, many trials still need to be made in order to ensure their efficiency.

The antioxidant, anti-inflammatory, and metabolic activities of marine terpenoids suggest that these compounds have potent and diverse protective effects against diabetes-induced vascular damage (10, 29). Marine terpenoids are superior to antidiabetic drugs that only address some of the metabolic characteristics that are altered in diabetes. However, the optimum dose and duration of marine terpenoids intake are still not available in humans and can be significantly different for each individual. Furthermore, clinical trials are necessary to determine the health benefits of marine terpenoids in human individuals with diabetes and the efficacy of the combined use of marine terpenoids with standard hypoglycemic therapy in relation to vascular protection in diabetes.

Hydroxylated essential oils and related marine terpenoids have potent antioxidant activity and can protect endothelial cells through direct and indirect effects. These include increased ROS scavenging, increased availability of NO, suppression of oxidized vascular components and the synergistic effects of other marine bioactive compounds (28). In light of their multi-target mechanism of action, marine terpenoids may protect against diabetes-related vascular damage beyond their ability to control glycemia. Clinical studies must assess their ability to improve glycemic control, vascular function, and clinical outcomes as compared to vascular-protective agents (33).

To conclude, the plethora of mechanisms by which marine terpenoids can directly regulate oxidative and inflammatory pathways has the potential to reduce the onset and progression of diabetes-induced vascular damage. Although these protective effects of marine terpenoids are very promising, some issues of bioavailability and clinical use must be further addressed to confirm their clinical relevance and to develop novel strategies for their therapeutic implementation in diabetic patients.

### 6.2 Neuroprotection (peripheral neuropathy, cognitive function)

Marine terpenoids are reported to mediate neuroprotective pathways in diabetes to treat both peripheral neuropathy and cognitive decline. Activation of the Nrf2/HO-1 pathway is one of the major mechanisms involved in the neuroprotective mechanism as it induces endogenous antioxidants, such as heme oxygenase-1, superoxide dismutase (SOD), and catalase (CAT) to inhibit neuronal damage by quenching reactive oxygen species (10). Further studies are needed to clarify the effect of marine terpenoids on the Nrf2/HO-1 pathway for long-term neuroprotection in chronic diabetic conditions.

Marine terpenoids decrease neuronal apoptosis and increase nerve cell survival in diabetic conditions by inhibition of oxidative stress and preservation of mitochondrial function (33). Although this has been reported, the role of each terpenoid in mitochondrial dynamics and energy metabolism in neurons has not been well addressed. They are also reported to possess anti-inflammatory activities by inhibiting NF-κB signaling, and thus reducing the expression of the pro-inflammatory cytokines TNF-α and IL-6 that contribute to the development of neuroinflammation in diabetic conditions (13). These activities could improve oxidative damage and preserve synaptic structure, leading to improved cognitive function. Further research is needed to determine if the same anti-inflammatory effects could be achieved in different diabetic neuropathy conditions using different doses of marine terpenoids.

Activating the Nrf2/HO-1 pathway by means of marine compounds to defend against diabetic neuropathy and cognitive decline could lead to the development of molecular-targeted intervention against these conditions that have limited effective antidiabetic treatments (10). Data from animal studies confirm that marine terpenoids attenuate oxidative damage and retain nerve structure in the central and peripheral nervous system, and that these neuroprotective activities are accompanied by decreased neuroinflammatory cytokines, oxidative damage markers, and neuronal degeneration (33). Marine terpenoid treatment significantly decreases the lipid peroxidation marker, malondialdehyde, in brain and peripheral nerve tissues, which has correlated with improved neurobehavioral assessments. More standardized experimental models are needed for further investigation to better understand the mechanisms of neuroprotection in both animal models and in humans.

Additionally, studies have demonstrated that marine terpenoids preserve myelin sheath and axon structure in diabetic neuropathy, mostly by reducing the oxidative balance and inhibition of the myelin and axon degradation pathways (41). Additional experiments are needed to confirm whether these compounds induce the remyelination process and nerve regeneration.

They are also able to significantly diminish astrocyte activation and glial cell proliferation *in vivo*, which is known to exacerbate neuroinflammation during the pathogenesis of diabetic neuropathy and cognitive decline (13). Marine terpenoids inhibit glial activation and therefore interrupt a neuroinflammation process that is a result of multiple pathways leading to glial activation and neuronal damage, including pro-inflammatory signaling, oxidative stress, and feedback between each signaling. However, deeper exploration into the effect of marine terpenoids on neuroinflammation in chronic diabetic conditions is warranted.

Histological examination reports that the nerve cells have been significantly protected against neuronal damage by marine terpenoids in chronic hyperglycemic conditions (33). Further evidence suggests that marine terpenoids possess neuroprotective activity against cellular damage in the nervous system via regulation of α-glucosidase and aldose reductase pathways, thereby suppressing the generation of advanced glycation end products (AGEs) and sorbitol associated with neuronal function dysfunction (41). Further studies are needed to confirm whether such dual inhibition can also be observed in human intervention studies to reduce diabetic neuropathy and cognitive decline.

Marine terpenoids suppress the postprandial blood sugar increase and thus could indirectly prevent the production of neurotoxic compounds by inhibiting glycation, which leads to glycated neural proteins and toxic sorbitol accumulation. It is hoped that these activities will improve overall neuronal function and attenuate the progression of cognitive deficits observed in diabetes (41).

By reducing the levels of aldose reductase that converts glucose into sorbitol, marine terpenoids suppress the sorbitol pathway in which sorbitol accumulation generates osmotic stress and oxidative damage that promotes diabetic neuropathy. Standard antidiabetic medication cannot suppress the flux of glucose toward this pathway (41). Further exploration is needed to establish their effect on the aldose reductase pathway at the clinical level as this enzyme also fulfills normal physiological functions in the human body.

The dual activities of regulating the glycemic level and the two major neurotoxic enzymatic pathways make marine terpenoids ideal candidates for neuroprotective intervention against diabetes, although further studies on the protective effects on each stage of the progress of diabetes and differences between type 1 and type 2 are needed (33). Further research into the synergistic action between these marine compounds and already available neuroprotective interventions should also be carried out.

By regulating redox-sensitive NF-κB signaling and downstream mediators, like pro-inflammatory eicosanoids that are synthesized through cyclooxygenase (COX) and lipoxygenase (LOX), marine terpenoids inhibit glial cell activation and neuronal apoptosis (39). COX and LOX metabolize arachidonic acid to produce prostaglandins and leukotrienes, which leads to the accumulation of inflammatory mediators in the diabetic central and peripheral nervous system (33). It remains uncertain whether these compounds are neuron-specific or whether their effects extend systemically.

Persistent activation of glial cells by oxidative stress, neuroinflammatory cytokines, and AGEs lead to neuropathy and cognitive deficits in diabetic conditions. Thus, one of the neuroprotective mechanisms is to shield neuronal function through multi-target regulation of these processes. It might be more effective than simply controlling a single pathway, and by combining these approaches, the need for higher concentrations of individual compounds could be reduced (39). Pharmacokinetic and pharmacodynamic studies are required to better understand the interaction of the target pathways of action with marine terpenoids.

Marine compounds improve neurotransmitter function, synaptic integrity, and the oxidative balance in nerve cells, suggesting that they possess neuroprotective functions against the pathogenesis of cognitive deficits in diabetics (30). Oxidative stress reduces neurotransmitter release in response to nerve stimulation, therefore protecting the neurotransmitter function will greatly improve the learning and memory process of diabetics to improve the quality of life. The majority of this information comes from animal experiments.

Marine terpenoids exhibit anti-inflammatory and antioxidative effects to preserve and stimulate synaptic plasticity and thus protect hippocampal neuron damage that plays a key role in the cognitive function (33). It is also suggested that marine terpenoids can preserve neuronal mitochondrial and neuronal energy metabolism to improve cognitive impairment in diabetic conditions (10). Studies focusing on human intervention are still lacking. Preserving the vascular function that leads to the improvement of the cerebral blood flow and reduction of endothelial injury helps to delay cognitive deficits in diabetic conditions and is one of the activities that marine terpenoids are believed to do (28).

**Figure 5:**
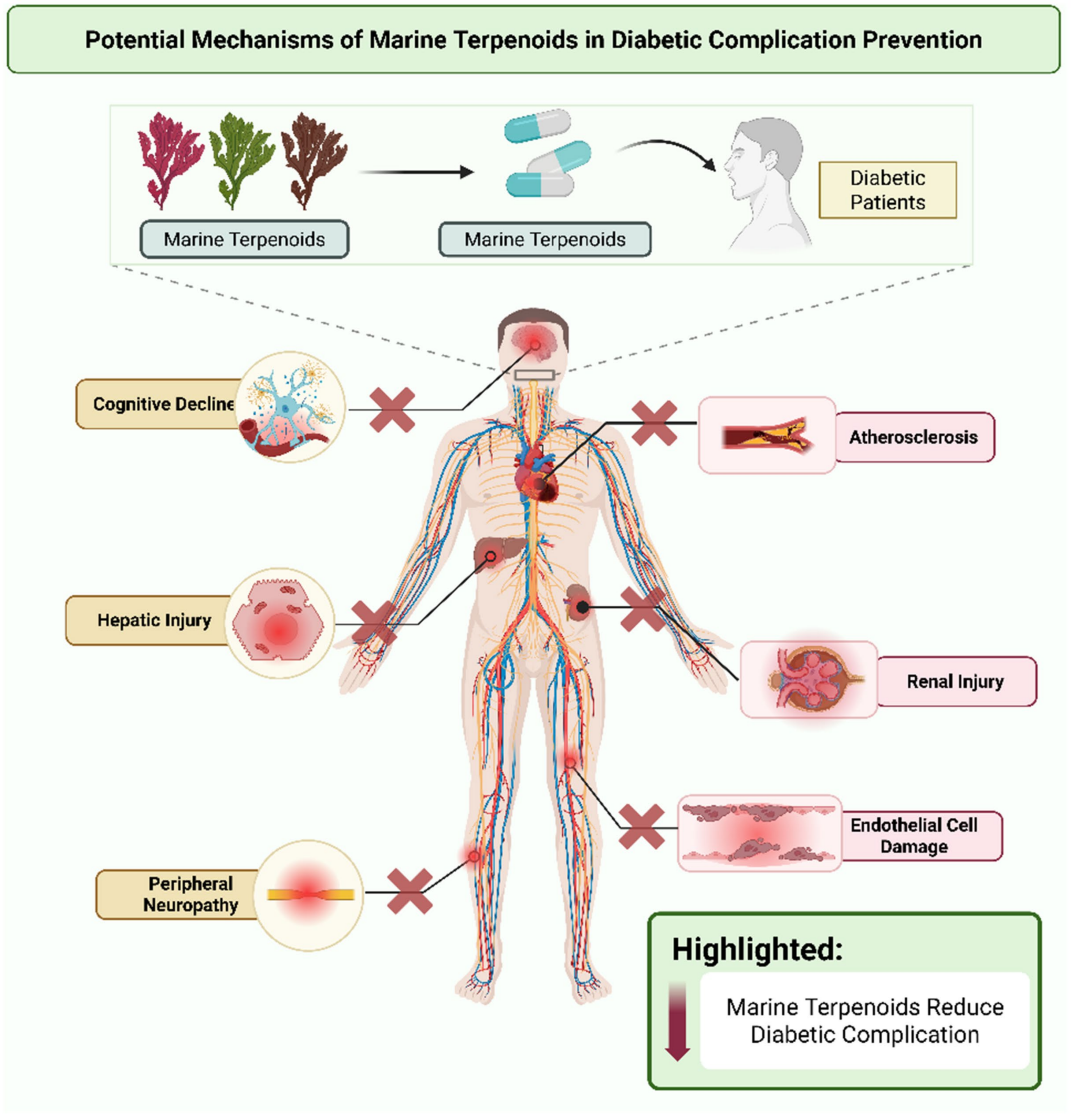
Schematic of diabetic complication prevention by marine terpenoids. Depicting their roles in protecting vascular, neural, renal, and hepatic tissues via redox and inflammatory pathway modulation.

Overall, marine terpenoids exhibit neuroprotective effects by reducing both oxidative and inflammatory pathways that can promote neuronal and neurovascular integrity in the brains of diabetics (Figure 5). However, further studies are required to assess whether these marine-derived molecules may prevent neuronal dysfunction in the long-term diabetic condition.

## 7 Translational potential and clinical perspectives

The pharmacokinetic behavior of marine terpenoids is very poor, leading to difficulties in clinical translation in terms of managing diabetes. This is due to their high molecular weight and hydrophobic nature which reduces intestinal absorption and distribution. Oral administration of marine terpenoids, therefore, has several drawbacks. Hence, new novel formulations which improve the bioavailability of these bioactive marine molecules through improved solubility, such as cyclodextrins, lipid-based systems, nanoparticles, and other delivery vehicles, may be required (43).

In addition, regulatory approval of marine terpenoids is highly dependent on their toxicity. *Padina tenuis*-derived extracts show little to no acute toxicity as determined *in vivo*. However, since thorough chronic toxicological tests are lacking, this poses a major hurdle in regulatory approval (43). Marine terpenoids also target many metabolic and signaling cascades, and therefore the effects of long-term administration may need to be determined, as well as possible detrimental effects on non-diabetic tissues and their overall safety in individuals with diabetes. Furthermore, these bioactive compounds have been shown to inhibit specific receptors, transporters, and channels which could have detrimental physiological roles in non-diabetic individuals. This can be determined by conducting a dose-escalation study and monitoring potential toxicity.

Metabolic stability of marine terpenoids poses yet another translational barrier for these bioactive marine molecules, as they are often similar to terrestrial bioactive molecules with low stability and limited efficacy in biological systems, which are rapidly eliminated (33). One major strategy to improve the therapeutic potential of marine terpenoids is to modify their structure or to embed them in microencapsulated matrices and biodegradable polymers to avoid rapid enzymatic degradation. This results in a longer half-life, which increases the availability of the bioactive marine molecules to the biological system. However, this requires more evidence-based preclinical studies, with optimized formulation strategies, prior to clinical application.

Interindividual variability can also result in the clinical development of marine terpenoids being difficult, due to the differences in metabolism based on species and differences in gut microbiome (44). A method to combat this may be to develop a more individualized medicine plan. For example, pharmacogenomics and microbiome manipulation may be able to optimize the response to marine terpenoids, however, these require additional studies.

Another obstacle hindering the therapeutic application of marine terpenoids is their limited availability and difficulty to synthesize. Marine sponges, corals, algae, and other marine organisms cannot be sustainably harvested for clinical application, and the chemical synthesis of bioactive marine compounds is limited due to their complex chemical structure (33). For this reason, aquaculture and the biological production of marine-derived chemicals are rapidly expanding to meet the demand for clinical application. It is critical to note that natural and synthetically/aquacultured bioactive marine compounds must be shown to have similar structures and equivalent activities for safe and effective implementation for clinical application.

Batch-to-batch variability of complex marine extracts is an obstacle in standard dosing and reproducibility in a clinical setting (33). This obstacle requires the development of marker-based quality control tests and green chemistry-based extraction methods. In addition, chemical complexity and limited reactivity may necessitate improvements to synthetic routes to yield efficient marine terpenoid synthesis. The environmentally friendly extraction of marine terpenoids is also a clinical application obstacle as it depends on the sustainable use of resources and environmentally friendly production practices. To do this, regulatory guidelines for the implementation of marine terpenoid extraction methods must be established in order to decrease the ecological footprint of commercial production. Thus, green extraction techniques are vital to ensure the sustainability and continued availability of these valuable compounds (33).

Although there is promising preclinical data showing the therapeutic value of marine terpenoids to treat diabetes, through mechanisms such as Nrf2/HO-1 pathway activation, to counteract oxidative tissue injury, there is still a paucity of published research in which the link between preclinical outcomes and their efficacy in humans is made (10). In addition, the translation from preclinical to clinical application also requires testing marine terpenoid bioactivity using human cells since rodent animal models of diabetes, in some cases, are unable to mimic the complex metabolic interactions of humans with diabetes.

In order to bridge this translational gap from preclinical to clinical application of marine terpenoids in diabetes management, several factors need to be considered and/or addressed. Firstly, clinical trials with controlled randomizations are needed, with endpoints that include: assessing glucose homeostasis, insulin sensitivity, and a decrease in the development of diabetes-related complications (44). Therefore, to develop evidence-based translational knowledge from the preclinical efficacy and *in vitro* studies of marine terpenoids for DM treatment, these novel therapies must be extensively studied with regard to their *in vivo* bioavailability, pharmacodynamics, drug metabolism, toxicity, as well as the clinical efficacy and safety to assess optimum dosing regimens. Without this approach, it may be difficult to provide an appropriate therapeutic dose and regime, and ultimately, the utilization of marine terpenoids will remain speculative.

A major challenge is that many marine terpenoids have poor pharmacokinetic behavior when administered orally. Their typically high molecular weight and lipophilicity translate to low water solubility and limited intestinal absorption (35). For instance, astaxanthin (MW ~596, very lipophilic) has an oral bioavailability in humans estimated between only 10–50% of a given dose (35), with variability depending on the formulation. Marine triterpenoids like fucosterol are even less soluble in aqueous environments, likely to result in minimal absorption unless taken with lipids. Rapid metabolism is another issue: being foreign bioactive, many marine terpenoids are substrates for phase I and II metabolism (e.g., oxidation by CYP450s, glucuronidation) which can lead to short half-lives and rapid elimination. For example, fucoxanthin is quickly metabolized to fucoxanthinol and other metabolites, which might have reduced activity.

To overcome these issues, novel formulations are required. Various strategies are being explored. For instance, using cyclodextrin inclusion complexes to improve water solubility, lipid-based systems (emulsions, self microemulsifying drug fucoery systems) to facilitate lymphatic absorption, and encapsulation in nanoparticles (polymeric nanoparticles, liposomes, or solid lipid nanoparticles) (35). These approaches have shown success in experimental settings. For instance, astaxanthin delivered in a nanoemulsion had significantly higher plasma levels in rats than astaxanthin powder (35). Chitosan-coated nanocarriers for astaxanthin improved their absorption and also provided a slow-release effect. Lipid nanoparticle encapsulation of fucosterol is being studied to enhance its delivery to target tissues (like the liver and pancreas) while protecting it from rapid metabolism. Such formulation advancements will be crucial for any clinical use, as an efficacious compound is irrelevant if it cannot reach therapeutic concentrations *in vivo*.

In addition, large interindividual variation in marine terpenoid bioavailability or pharmacological response in different patient populations will greatly influence the development of these potential therapies in DM. As several marine terpenoids have shown the ability to regulate glucose metabolism in preclinical studies (31), it is important to determine which individuals may respond positively, negatively, or indifferently to marine terpenoids. Patient variability may arise from various sources, including, but not limited to, sex, genetics, gut microbiome, presence of diabetes-related comorbidities, and polypharmacy. In the clinical arena, personalized medicine could provide a targeted marine terpenoid treatment for these patient populations. This would need in-depth patient profiling regarding their genetic, metabolomic, and microbiotic components to assess specific individual responses to marine terpenoids.

Regulatory approval will heavily depend on demonstrating safety. Initial indications are that many marine terpenoids have low acute toxicity. For example, extracts from Padina (rich in terpenoids) showed little to no acute toxicity in rodents up to high doses (42, 43). Astaxanthin is widely consumed as a supplement with an excellent safety profile (no significant adverse effects up to 40 mg/day in humans in trials). However, chronic toxicological tests are lacking for most compounds (35). Potential concerns include: (i) Off-target effects due to multi-target nature, which is the long-term administration might subtly affect non-diabetic pathways. For instance, if a marine terpenoid blocks a certain ion channel or receptor at high doses, it could lead to unanticipated effects in other tissues. (ii) Bioaccumulation, which for some marine terpenoids might accumulate in fatty tissues given their lipophilicity, raise questions about long-term deposition and any possible toxicity from metabolites (35). (iii) Interactions with other medications, which since diabetic patients often take multiple drugs (metformin, statins, antihypertensives), any new therapy.

A specific example of a safety consideration: immune modulation. If a compound strongly suppresses NF κB, could it impair immune responses over time? Possibly, though in diabetic contexts this might actually be beneficial (reducing chronic inflammation). Still, dose-escalation studies in animals and careful monitoring in any human trials are necessary to detect such issues (35). Conducting maximum tolerated dose (MTD) studies and chronic (6+ months) toxicity studies in at least two species will be important steps. These will assess potential organ toxicities (hepatic, renal, etc.), carcinogenicity (unlikely given these are not DNA reactive, but standard to check), and effects on reproductive health. Beyond intrinsic toxicity, another critical aspect of safety evaluation involves understanding how marine terpenoids interact with existing antidiabetic medications, particularly given their metabolic clearance pathways and potential for pharmacodynamic overlap.

There are few published studies on marine terpenoids which show how they will interact with currently prescribed oral anti-diabetic drugs. Many novel marine terpenoids that regulate glucose metabolism may be cleared by the liver or kidney. Therefore, to ensure their safe and effective implementation in the clinical application, drug interactions are crucial to evaluate for any potential synergism or antagonism in DM (41).

With any new drug on the horizon, several factors are assessed, and regulatory policies are created. A number of regulatory agencies, such as the Food and Drug Administration (FDA), Health Canada, and the World Health Organization (WHO), exist for new pharmaceuticals, including the regulatory approvals for marine natural products, to make sure that the products being offered are safe and efficient. At the current time, no standardized regulatory guidelines exist for marine terpenoid therapeutics in DM, meaning that several issues are still of concern for patients and physicians (33). This includes the quality of marine-derived therapies, potential drug–drug interactions, potential adverse effects, and long-term safety issues. Therefore, marine terpenoid-based pharmaceuticals are far from market clearance for implementation in DM, and several issues need to be addressed, beginning with formal guidelines on product specifications, testing for quality control, quality assurance testing and safety standards, clinical trial design and management, and post-marketing surveillance. In order to successfully achieve clinical approval, an international coordinated effort is required that involves physicians, scientists, as well as regulatory bodies, to establish clear guidelines for safe clinical administration of these novel therapeutics.

One of the main difficulties in the therapeutic application of marine terpenoids is how to incorporate them into conventional clinical management in DM. The development of marine terpenoids as a new medication requires a team consisting of pharmacology, marine biotechnology, and clinical expertise, to ensure these compounds will be developed to be both safe and efficient as novel therapies (44).

There is considerable data from experimental and/or preclinical models demonstrating that marine terpenoids show efficacy and exhibit safety and clinical applicability better than those found in currently marketed oral anti-diabetic drugs. To strengthen the justification for marine terpenoids being potential clinical therapeutics, comparative studies of efficacy versus currently available anti-diabetic drugs, such as the gold standard oral anti-diabetic agent metformin, must be performed in individuals with DM (41, 44). By bridging this translational gap and ensuring that they demonstrate the desired clinical effectiveness and safety with minimal side effects or toxicities, marine terpenoids can be implemented in modern clinical treatments for DM. Despite strong preclinical support, the clinical development of marine terpenoids for diabetes remains in its early stages, with few compounds progressing into human trials.

As of now, no marine terpenoid is an approved antidiabetic drug, and clinical trials have been relatively rare (45). According to a recent review, numerous marine compounds are under clinical investigation in general, but for T2DM specifically the trials are limited, and none have advanced to market (45). Notably, that review mentioned around 20 marine compounds in Phase I–III trials for various diseases, but for T2DM, fucoidan and brown seaweed extracts have seen small trials, and marine collagen peptides and algal polysaccharides have been in some trials (45). There are several highlights on the clinical trials as follows:

- An oral fucoidan trial in obese non-diabetic adults showed no significant effect on glycemic control (no changes in fasting glucose or insulin) (45). Another in normoglycemic subjects found no effect on postprandial glucose (45). These null results suggest fucoidan (a sulfated polysaccharide, not a terpenoid but from same source) might not help unless in a specific prediabetic population. Indeed, an ongoing trial is testing fucoidan in pre-diabetics (ACTRN12621000413820) (45), which will clarify if a more at-risk group benefits.
- A clinical trial of a dieckol-rich *Ecklonia cava* extract in prediabetic individuals showed positive results. It reduced blood glucose and insulin resistance without side effects (45). This is very encouraging and indicates that at least some marine-derived compounds can be efficacious in humans. However, since the extract contained multiple components (phlorotannins mainly), it’s unclear how much terpenoids contributed. Still, it demonstrates proof-of-concept that marine natural products can translate to clinical benefits.
- A trial of marine collagen peptides in type 2 diabetic patients found improvements in fasting glucose, HbA1c, insulin, and lipid levels (45). Collagen peptides aren’t terpenoids, but this speaks to the broader potential of marine nutraceuticals in metabolic disease.

From a regulatory standpoint, currently no formal guidelines exist specifically for marine terpenoid based therapeutics in DM (45). Agencies like FDA and EMA will treat them as any new chemical entity, requiring demonstration of quality, safety, and efficacy. One challenge is the quality control of natural product mixtures: regulators prefer defined active ingredients. Thus, isolating the active terpenoid or having a standardized extract is critical. The absence of guidelines also means innovators need to work closely with regulators to define acceptable specifications and trial designs. Given these regulatory expectations, the development of a standardized and well-characterized marine terpenoid formulation becomes a foundational prerequisite for clinical translation.

To bring a potential novel marine terpenoid from laboratory to clinic as a novel medication will require that a high-quality standardized marine terpenoid preparation has to be made and characterized. These high standards ensure quality control and consistency for clinical trials and product launch (33). These improvements can be made by investment from academic and industrial collaborations.

In summary, the path to the therapeutic application of marine terpenoids is still a long way from being complete. Although these marine terpenoids have shown therapeutic promise, with potential to manage diabetes, these marine natural compounds will need to address a number of critical translational obstacles before these novel molecules can achieve therapeutic application in humans.

## 8 Conclusion

Marine terpenoids represent a valuable resource for combating diabetes-related oxidative stress, offering novel therapeutic strategies through their multifaceted antioxidant and antidiabetic activities. By addressing their unique advantages as well as challenges (e.g., bioavailability, sustainability), this review provides a comprehensive and up-to-date synthesis of the field. Preclinical findings provide robust evidence for their potential to protect pancreatic β-cells, enhance glycemic control, and prevent secondary complications such as vascular and renal damage. However, clinical translation remains limited due to challenges in bioavailability, consistent extraction, formulation strategies, and comprehensive safety profiles. Future research must focus on overcoming these translational barriers through interdisciplinary collaboration, optimized extraction and formulation techniques, and rigorous human trials. Addressing these aspects could position marine terpenoids as a significant advancement in the treatment and management of diabetes and its associated complications.
